# Natural resorcylic lactones derived from alternariol

**DOI:** 10.3762/bjoc.20.187

**Published:** 2024-08-30

**Authors:** Joachim Podlech

**Affiliations:** 1 Karlsruhe Institute of Technology (KIT), Institute of Organic Chemistry, Kaiserstraße 12, 76131 Karlsruhe, Germany

**Keywords:** biosynthesis, fungal metabolites, polyketides, resorcylic lactones, total synthesis

## Abstract

In this overview, naturally occurring resorcylic lactones biosynthetically derived from alternariol and almost exclusively produced by fungi, are discussed with view on their isolation, structure, biological activities, biosynthesis, and total syntheses. This class of compounds consists until now of 127 naturally occurring compounds, with very divers structural motifs. Although only a handful of these toxins (i.e., alternariol and its 9-*O*-methyl ether, altenusin, dehydroaltenusin, altertenuol, and altenuene) were frequently found and isolated as fungal contaminants in food and feed and have been investigated in significant detail, further metabolites, which were much more rarely found as natural products, similarly show interesting biological activities.

## Introduction

**Figure 1:**
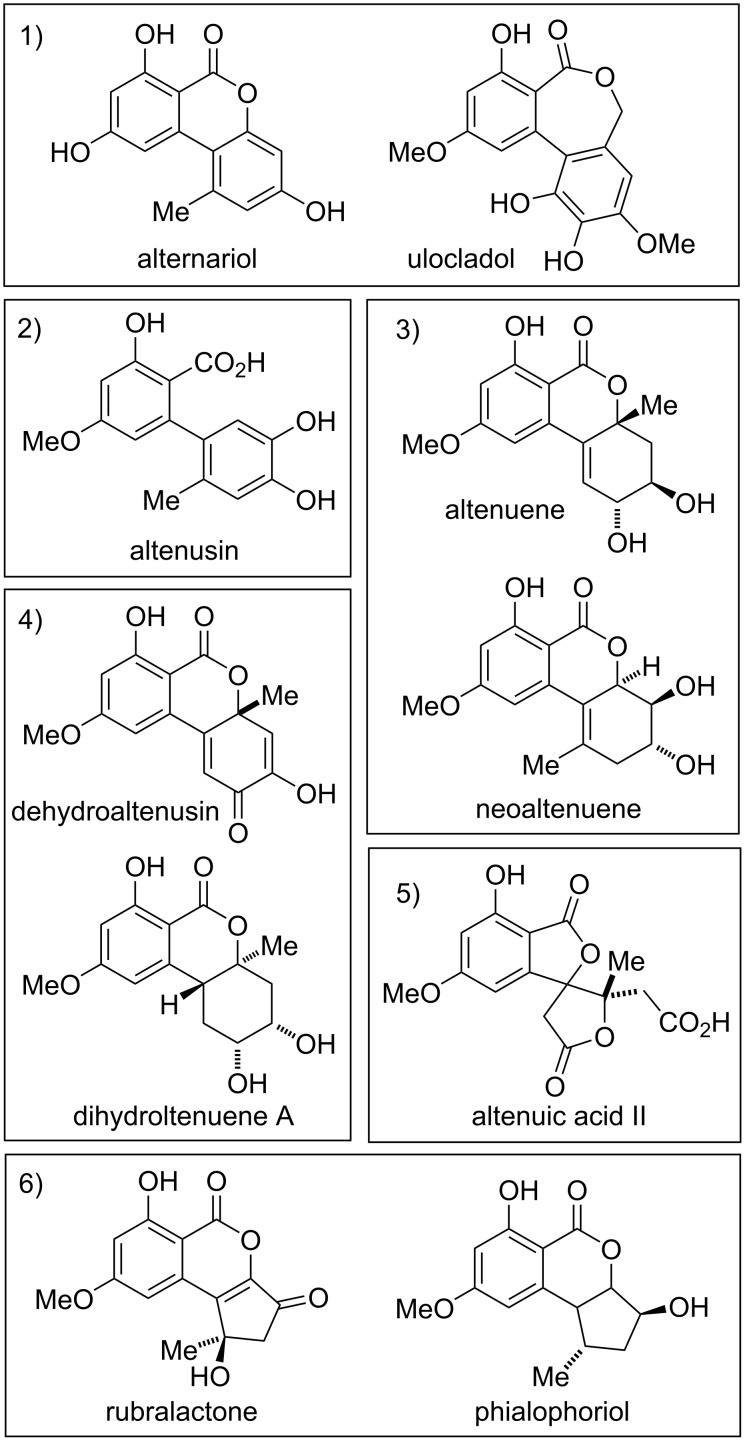
Examples of compounds covered in this review categorized in six sub-classes (see text).

Alternariol and some of its derivatives are ubiquitous as fungal metabolites present in infested plants and in food and feed, but similarly in soil, in wallpapers, and in textiles. Although the parent alternariol and the plethora of compounds biosynthetically derived from alternariol are very diverse and show numerous detrimental but also beneficial biological properties, they have never been comprehensively surveyed in a review. Nevertheless, quite a number of overviews exist on mycotoxins in general [1–3], on selected Alternaria toxins [4–12], and on dibenzo-α-pyrones [13–19]. The current review will comprehensively deal with all naturally occurring polyketides derived from β-resorcylic acid (2,4-dihydroxybenzoic acid), whose biosynthesis is presumably starting from alternariol. The lactone moieties of these compounds are usually six-membered rings, where variations during or after polyketide synthesis occasionally give rise to five- or seven-membered rings or even to open structures with a free resorcylic acid. Derivatives formed through metabolization in the human body (or in animals) are only covered if the respective metabolites were similarly identified as natural products. A thorough survey of the literature revealed (at now) 127 natural products to be classified as natural resorcylic lactones derived from alternariol. These will herein be categorized in six sub-classes (Figure 1):

1. alternariol and its substituted derivatives (46 members),
2. biaryls (7 members),
3. altenuene and its diastereomers and derivatives (20 members),
4. oxidized and reduced altenuenes (21 members),
5. altenuic acids and related compounds (7 members), and
6. cyclopenta-fused derivatives (26 members).

These compounds will be strictly organized due to their structures and not due a possible concurrent isolation or even to common names (e.g., graphislactones A, C, E, and G will be treated in different sub-chapters, although graphislactones A and C were isolated together and all of them share a common name).

**Figure 2:**
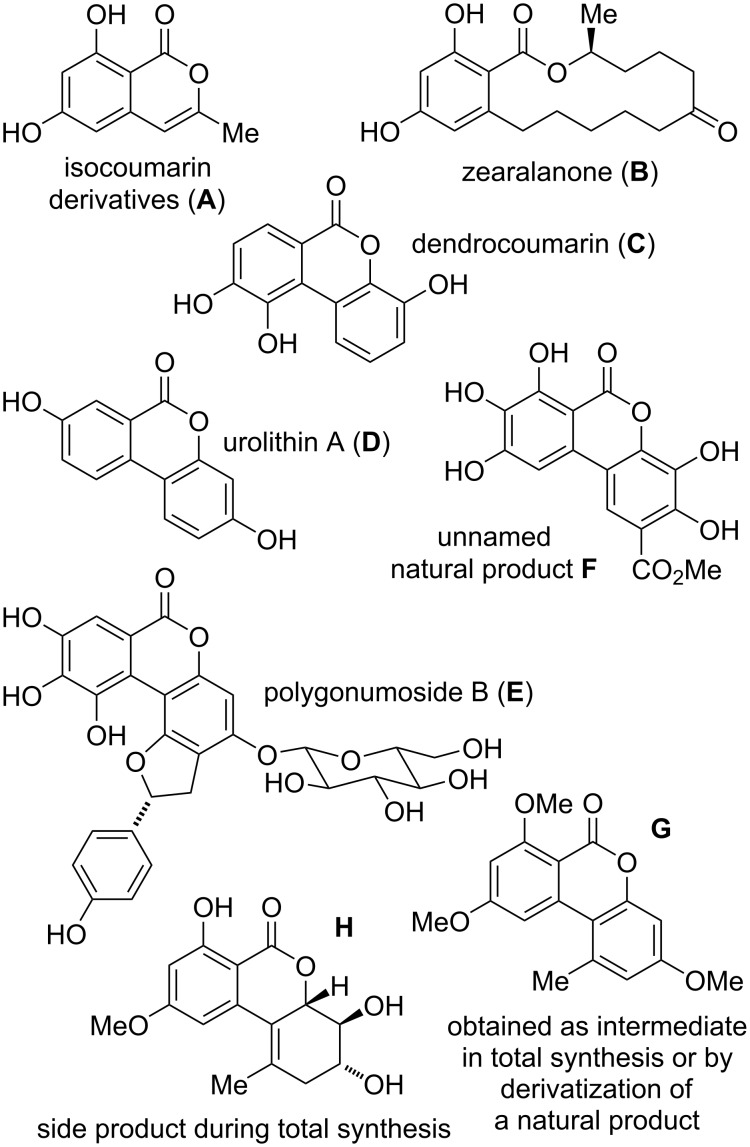
Examples of compounds not covered in this review.

Since it is intended to focus on naturally occurring alternariol-derived compounds, a number of somewhat related structures will not be discussed herein: Isocoumarins [20–21] and other structures, which are most likely not derived from alternariol (e.g., **A**, Figure 2), will not be included. Resorcylic lactones [22–24] structurally not related to alternariol-derived dibenzo-α-pyrones, like zearalenone (**B**), are similarly not part of this review. These types of resorcylic lactones could be easily differentiated by taking the involved polyketide synthase (PKS) into account [25], since polyketides like zearalenone are synthesized by means of a type I PKS [22,26], while alternariol and its derivatives are likely to be obtained by catalysis with a PKS of type II [27] or possibly of type III [28]. However, no reliable information in this respect seems to be available for alternariol or even for its derivatives. Compounds not containing a fully intact resorcylic acid (e.g., dendrocoumarin (**C**) [29], urolithin A (**D**) [30], or polygonumoside B (**E**) [31]) or containing more than the two hydroxy groups of the resorcylic acid (e.g., unnamed natural product **F** [32]) will not be discussed (with scarce exceptions, when the respective compounds are most likely derived from alternariol or from related natural products). Compounds, which have not been isolated as natural products, but are synthetic derivatives [33] (e.g., **G**) of natural products, or intermediates [34] (e.g., **G**) or side products [35] (e.g., **H**) during their total synthesis, are neither systematically covered in this review.

**Figure 3:**
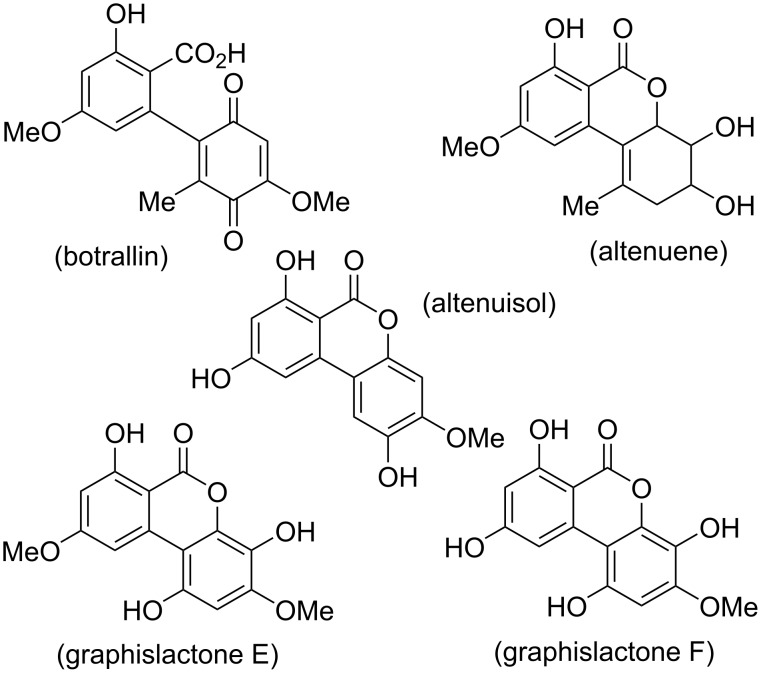
Wrongly assigned and thus obsolete structures (details will be discussed in the respective chapters).

A number of wrongly assigned structures have been proposed over the decades (Figure 3). These are mentioned here in summary, where further details will be given in the respective chapters: A quinone structure has been given with the first report of botrallin [36], but was corrected some years later [37]. A wrong constitution has originally been proposed for altenuene [38]; it was revised shortly after [39]. The originally proposed structure [40] of altenuisol is wrong: Total synthesis and comparison of the NMR spectra revealed that this natural product is identical with altertenuol [41], whose structure was correctly given previously [42]. Furthermore, the originally proposed structures [43] of graphislactones E and F were corrected after total synthesis and comparison of NMR spectra [44].

Occasionally, natural products have been reported more than one time as new compounds and were given several names. Only the first given name should be used and further names are thus obsolete: Verrulactone D is erroneously given in the SciFinder database as synonym for altertenuol, but is in fact a different natural product. The originally proposed structure of altenuisol turned out to be wrong; it is in fact identical with the structure of altertenuol and the name ‘altenuisol’ is thus obsolete (although it is still frequently used in the community) [41]. The occasionally used designations ‘graphislactone S1–S4’ were used in the initial reports [45–46], but have not been proposed as names for these compounds. The names ‘graphislactones A–D’ were used in the following reports by the authors and by most of the community. The name ‘talaroflavone’ has once erroneously been assigned to 1-deoxyrubralacton (**116**) [47]. Unfortunately, this mistake has been adopted in a later report and the names ‘deoxytalaroflavone’ (for **106**) and ‘7-hydroxy-deoxytalaroflavone’ (for **107**) were proposed [48], although these compounds have no structural relationship with talaroflavone (**122**) and furthermore the name ‘deoxytalaroflavone’ had already previously been assigned to a different compound **110** [49]. The name ‘7-hydroxy-deoxytalaroflavone’ is misleading and thus should not be used.

The most prominent and most frequently referenced members of the herein discussed class of natural products are the parent alternariol (**1**, 1099 publications given in the SciFinder database at 04/2024), 9-*O*-methylalternariol (**2**, 578 entries), altenuene (**54**, 320), altenusin (**47**, 130), dehydroaltenusin (**74**, 55), altertenuol (**31**, 45), and isoaltenuene (**55**, 24), where the most abundant Alternaria toxins are usually abbreviated: Alternariol (AOH), 9-*O*-methylalternariol (AME), altenuene (ALT), and altenusin (ALS) have commonly accepted abbreviations, while further abbreviations are used inconsistently (e.g., iALT or isoALT for isoaltenuene).

*Alternaria* metabolites [7,9–11] (which not only consist of the herein discussed resorcylic lactones) are predominantly, but by far not exclusively, isolated from *Alternaria* spp., especially from *Alternaria alternata*. The genus *Alternaria* in the family Pleosporaceae (Pleosporales, Dothideomycetes, Ascomycota) belongs to the fungi imperfecti and all species are known as plant pathogens [50–52]. It should be noted that *Alternaria alternata* is a species complex and many producer strains reported in the literature were not correctly identified [53]. The rather low toxicity of alternariol and most of the further *Alternaria* toxins (especially those with resorcylic lactone structure) discouraged a thorough investigation of their biological activities, especially of their toxicities. The European Food Safety Authority (EFSA) stated that “at present (2011), the knowledge on the possible effects of *Alternaria* toxins on farm and companion animals as well as the database describing the occurrence of these mycotoxins in feedstuffs are scarce and are not sufficient to assess the risk regarding *Alternaria* toxins for animal health,” [9] and encouraged further investigations on *Alternaria* toxins in many areas.

## Review

### Alternariol-derived resorcylic lactones

#### Alternariol and its substituted derivatives

**Figure 4:**
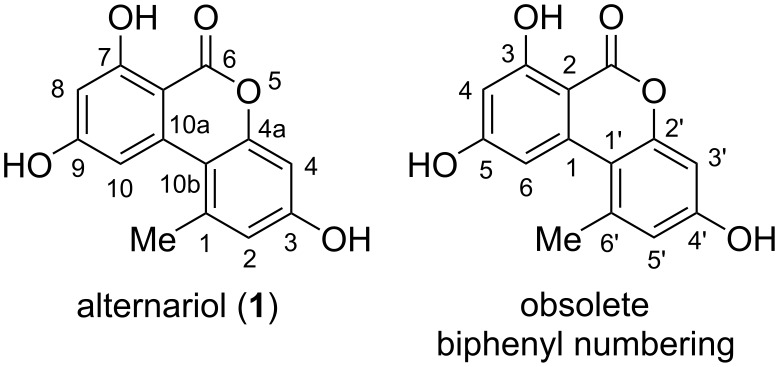
Alternariol with the correct IUPAC numbering and an occasionally used numbering based on the biphenyl substructure.

**Alternariol:** Alternariol (AOH, **1**, Figure 4) was first isolated in the early 1950ies from *Alternaria tenuis* (synonymous with *A. alternata*). Its structure was elucidated with chemical analysis methods [33,54] and later unambiguously confirmed by X-ray crystallographic analyses [55–57]. Its biosynthetic origin from acetate units by feeding 1-^14^C-labeled acetate precursors was recognized soon after [58–59], where more details on its biosynthesis are given in the respective chapter (vide infra). Alternariol is the main toxin produced by *A. alternata* and was isolated from further *A.* spp. (i.e., *A. longipes* [60], *brassicae*, *capsica-anui*, *citri, cucumerina*, *dauci*, *kikuchiana*, *solani*, *tenuissima*, and *tomato* [6]) and from numerous further fungal genera. Various total syntheses have been presented over the decades [34,61–64] including biomimetic syntheses [65–68] and syntheses of labeled alternariol [69–70]. Selected syntheses will be given in the chapter on total syntheses (vide infra).

The ubiquity and easy availability of this toxin by microbiological and synthetic methods (5 g of the compound were once synthesized in the group of the author) allowed for numerous biological investigations, which can hardly be summarized comprehensively in this overview, but can be further studied in numerous reviews on this and related toxins [6,10–11,15,71–72].

Already in the first reports on alternariol, an antibacterial activity against *Staphylococcus aureus* (at 25 ppm) and *Escherichia coli* (at 50 ppm) was mentioned [33], which was later corrected to an activity against the Gram-positive *S. aureus* and *Corynebacterium betae*, but not against Gram-negative *E. coli* [73]. It was further noted that AOH showed neither fungicidal (possibly due to its low solubility in the standard tests, performed in aqueous media) nor phytotoxic activity [73].

A general but not an acute toxicity of AOH has already been mentioned in late 1970ies, e.g., an activity against *Bacillus mycoides* at 60 μg/disc and a toxicity to HeLa cells with an ID_50_ value of 6 μg/mL [74]. It turned out that induced cytotoxicity [75] is mediated by activation of the mitochondrial pathway of apoptosis in human colon carcinoma cells [76–77] and that cytotoxicity on HCT116 cells is increased, when AOH is combined with 9-*O*-methylalternariol (**2**, AME) [78]. AOH further showed to have a detrimental effect on initial embryo development [79]. Further reports on its mutagenicity and genotoxicity have been published in the due course [80–83], but it seems as if an unambiguous mutagenic activity has not been proven before 2006, when AOH was found to cause mutations in cultured Chinese hamster V79 cells and in mouse lymphoma cells even at non-toxic or moderately cytotoxic concentrations [84]. AOH was further shown to cause strand-breaking in mammalian cell lines V79, HepG2, and HT-29 [85–87]. It was a significant finding by Marko et al. that AOH acts as a topoisomerase poison, preferentially affecting the IIα isoform [88], where on the other hand the damage turned out to be repaired in less than two hours [89]. Its induction of oxidative stress leading to DNA damage [90–93] and its causing cell cycle arrest, apoptosis, and changing the cell morphology further contribute to the detrimental effects of AOH [94–96]. AOH furthermore activated the nuclear translocation of the aryl hydrocarbon receptor (AhR) and the nuclear factor erythroid 2-related factor 2 (Nrf2) [97]. The possible anticancer effects of AOH through its cytotoxic, antiinflammatory, genotoxic, mutagenic, apoptotic, and anti-proliferative effect and its influence on immune response [98], cell cycle, and autophagy have been comprehensively summarized only recently [17].

AOH has been found to be a weak estrogenic mycotoxin that also has the ability to interfere with the steroidogenesis pathway [99], to have a negative effect on progesterone synthesis in porcine granulosa cells [100], and to be an androgen antagonist with an EC_50_ value of 269 μM [101].

Phase I [102–103] and phase II [104–106] metabolization of AOH has been thoroughly investigated and it turned out that AOH is not metabolized by human fecal microbiota [107].

**Figure 5:**
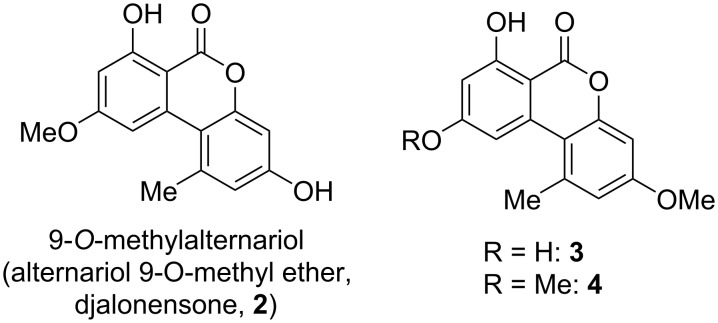
Alternariol *O*-methyl ethers.

**Alternariol methyl ethers:** Methyl ethers of alternariol have been obtained during chemical structure elucidation and as intermediates in total syntheses of natural products, but some of these have been furthermore isolated as natural products (Figure 5). First to mention is 9-*O*-methylalternariol (**2**), mostly referred to as alternariol 9-methyl ether, usually abbreviated with AME, and occasionally named djalonensone. It is almost as abundant as alternariol itself and it is hardly possible to catch up with the number of publications on this toxin; the SciFinder database gives at now more than 500 entries to this compound. It was first isolated together with alternariol from *Alternaria tenuis* (synonymous to *A. alternata*) [33,54]. Its correct structure was proposed, based on chemical methods, shortly after [42] and was later unambiguously confirmed by total synthesis [62] and finally by an X-ray crystallographic analysis [108]. Further syntheses have been presented over the decades including a biomimetic synthesis [68] and a synthesis of deuterated AME [70]. It was isolated from numerous fungal sources, especially from *Alternaria* spp. (i.e., *A. brassicae*, *capsici-anui*, *citri*, *cucumerina*, *dauci*, *kikuchiana*, *longipes*, *porri*, *solani*, *tenuissima*, and *tomato*) [6]. When it was further isolated from the small tree *Anthocleista djalonensis*, the authors considered it to be a new natural product and proposed the name ‘djalonensone’, which is occasionally used in the literature [109]. AME has been investigated thoroughly on its biological activities, especially on its toxicity [6,12]. Its phytotoxicity has already been noted in the first reports; it induced chlorosis when injected to the leaves of tobacco plants [110]. It turned out to be cytotoxic against HeLa and lymphoma L5178Y cells with ID_50_ values of 8 μg/mL [74], was a strong mutagen in *Escherichia coli* strain ND-160 [111], was maternally toxic and fetotoxic to Syrian golden hamsters [112], induced mitochondrial apoptosis in human colon carcinoma cells [113], induced cytochrome P450 1A1 formation and apoptosis in murine hepatoma cells dependent on the aryl hydrocarbon receptor [96], and significantly increased the rate of DNA strand breaks in human carcinoma cells (HT29, A431) at concentrations ≥1 μM [88]. A reported mutagenicity against *Salmonella typhimurium* strains TA98 [80] was later revised and attributed to contamination with the strongly mutagenic altertoxins [82–83]. When administered to rats, AME induced gene mutations, chromosome breakage, and DNA damage [114]. AME turned out to be active against bacteria (*Bacillus subtilis*, *Staphylococcus haemolyticus*, *Agrobacterium tumefaciens*, *Pseudomonas lachrymans*, *Ralstonia solanacearum*, and *Xanthomonas vesicatoria*) with IC_50_ values ranging from 16 to 38 μg/mL, showed antinematodal activity against *Caenorhabditis elegans* and *Bursaphelenchus xylophilus* (IC_50_: 74.6 and 98.2 μg/mL, respectively), and suppressed spore germination of the fungus *Magnaporthe oryzae* (IC_50_: 87.2 μg/mL) [115]. It furthermore had a negative effect on progesterone synthesis in porcine granulosa cells [100] and selectively inhibited human monoamine oxidase-A (MAO-A) with an IC_50_ value of 1.71 μM (but did not inhibit MAO-B) and was thus considered for the treatment of depression, Parkinson’s disease, and Alzheimer’s disease [116]. Its metabolization in phase I [102–103,117] and phase II [104,118–120] has repeatedly been investigated. It turned out that it is not metabolized by human fecal microbiota [107]. Protocols for its standardized analysis in food and feed have further been developed [121–122].

3-*O*-Methylalternariol (**3**) was isolated as a natural product only once from a non-specified *Alternaria* sp. [123] and its structure was confirmed by comparison with an intermediate obtained during the total synthesis of alternariol [66].

3,9-*O*,*O*-Dimethylalternariol (**4**) was first isolated from an unidentified endophytic fungus [124] and later from *colletotrichum* sp. [125], *Lachnum abnorme* [126], *Diaporthe phragmitis* [127] and a further *D.* sp. [128], and deviantly from the flower buds of the banana species *Musa nana* [129]. Its structure was determined by NMR spectroscopy [126] and unambiguously confirmed by independent synthesis [130]. It showed inhibitory activity against *Pseudomonas syringae* pv. *actinidae* with an MIC value of 25 μg/mL [127].

***O*-Glucosides,* O*-acetates, and *O*-sulfates of alternariol:** As for all natural products bearing hydroxy groups, especially of phenolic hydroxy groups [131–133], it is quite likely that alternariol and its derivatives are partly present in the organisms as *O*-glucosides, acetates, or sulfates [134–135], where these substituents are most generally cleaved during the isolation and work-up processes. These derivatives (glucosides and sulfates) are furthermore built during phase II metabolization in the body [120,122,136–137], where the respective metabolites are not comprehensively covered in this overview.

**Figure 6:**
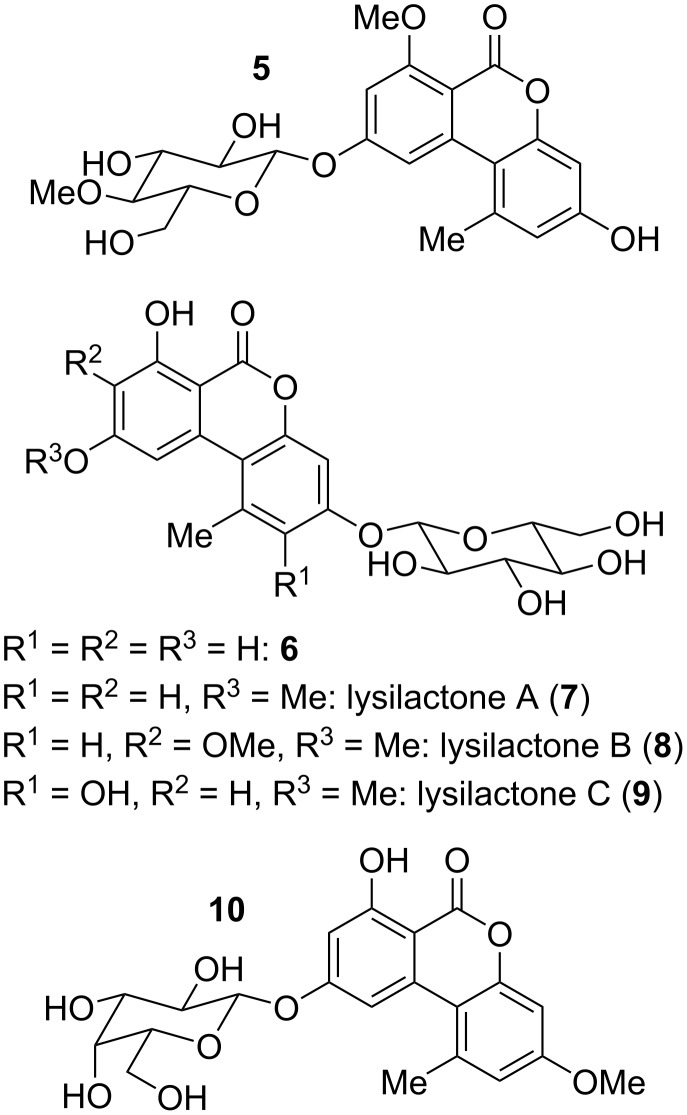
Alternariol *O*-glycosides.

A small number of glucosides bearing alternariol derivatives have been isolated as natural products (Figure 6). 7-*O*-Methylalternariol 9-*O*-β-[4-methoxylglucopyranoside] (**5**) was isolated from *Alternaria alternata* and its structure was determined by NMR spectroscopy. It showed no antibacterial activity [138].

Lysilactones A–C (**7**–**9**) bearing β-glucopyranoside moieties were first isolated from *Lysimachia clethroides*, where lysilactone B is mentioned in this review only due to its structural analogy to the other lysilactones; it is not a resorcylic lactone and has not been mentioned after the initial report [139]. The structures of these compounds were determined by NMR-spectroscopic methods and constitution and configuration of lysilactone A was further unambiguously confirmed by total syntheses [139–141].

β-ᴅ-Galactopyranoside **10** was isolated from *Penicillium* sp. [142], where it turned out to be cytotoxic against A559 cells (IC_50_: 6.8 μg/mL) but not against further tested cell lines.

Alternariol-derived glucopyranoside **6** was identified together with lysilactone A in LC–MS/MS analyses of invested cereals based on comparison with synthesized samples [141], but seems to have never been isolated or further investigated.

**Figure 7:**
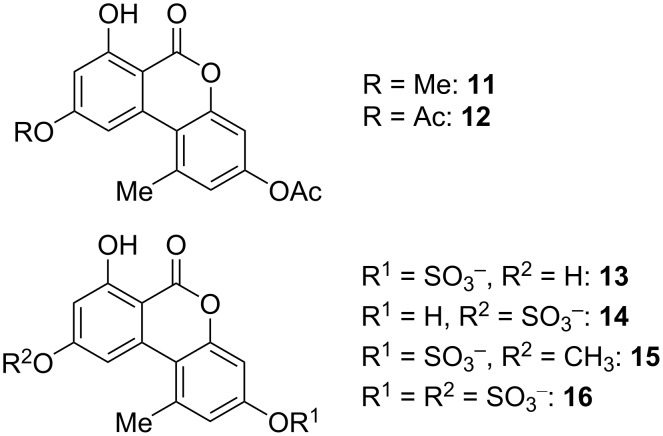
Alternariol *O*-acetates and *O*-sulfates.

3-*O*-Acetyl-9-*O*-methylalternariol (**11**) was isolated from an unidentified endophytic fungus [124] and 3,9-*O*,*O*-diacetylalternariol (**12**) was found in *Diaporthe cameroonensis* [143]. No biological activities were determined for these compounds (Figure 7).

A number of sulfate conjugates have been synthesized and used as standards for mass-spectrometric or HPLC analyses [134,141,144]. In this course it turned out that 3-*O*-sulfates of alternariol and 9-*O*-methylalternariol and the alternariol-9-*O*-sulfate (**13**–**15**) are conjugates repeatedly present in fungal sources [134,141,145] and the 3,9-*O*,*O*-disulfate of alternariol (**16**) has similarly been isolated [146]. Due to the instability of these sulfates towards hydrolysis they were typically not isolated. Nevertheless, alternariol sulfates were isolated from *Alternaria* sp. and alternariol-9-*O*-sulfate (**14**) turned out to be active against 23 protein kinases with IC_50_ values ranging from 0.22 to 8.4 µg/mL and to be cytotoxic against L5178Y cells (EC_50_: 4.5 µg/mL) [147]. The sulfated 9-*O*-methylalternariol derivative **15** was isolated from *A. alternata* and showed inhibitory activity against HCV NS3/4A protease (IC_50_: 52.0 μg/mL), cytotoxic activity against HEP-G2 cancer cells, and turned out to be antibacterial against *Bacillus cereus*, *B. megaterium*, and *Escherichia coli* with inhibition zones of 17, 12, and 10 mm, respectively, at 50 μg/disk [148]. There is some evidence that not only alternariol and its 9-*O*-methyl ether can be present as sulfated conjugates, but that further toxins could similarly be sulfated in the organisms, e.g., altenusin and dehydroaltenusin [149].

**Figure 8:**
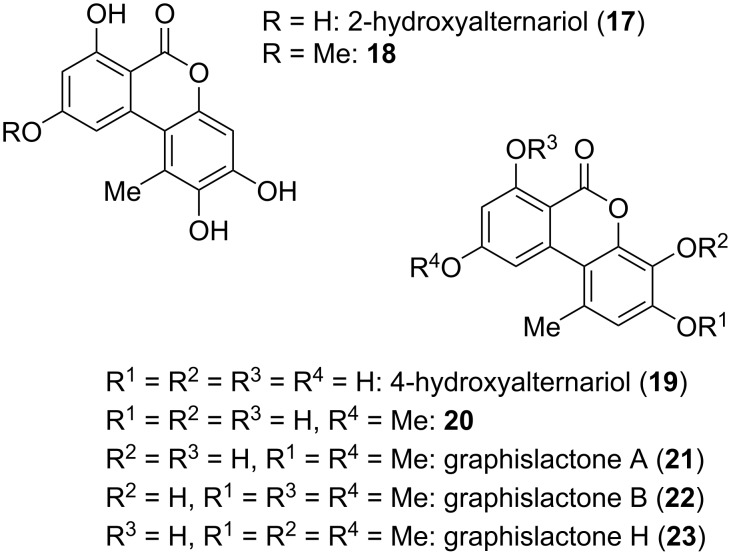
2-Hydroxy- and 4-hydroxy-substituted alternariol and its *O*-methyl ethers.

**Substituted alternariol and the respective O-derivatives:** 2-Hydroxyalternariol (**17**) and the respective 9-*O*-methyl derivative **18** were identified in the human, rat, and porcine metabolism [102–103,150], but **17** was later similarly isolated as a fungal natural product from *Diaporthe* (*Phomopsis*) sp. [128,151–154] and *Alternaria* sp. [28,47,154] (Figure 8). It showed antioxidant activity with an IC_50_ value of 42.8 μM [153], inhibited about 90% of iNOS (inducible nitric oxide synthase) expression when applied at 20 μM, decreased the protein expression levels of pro-inflammatory cytokines (tumor necrosis factor-α, interleukin-6, and monocyte chemotactic protein 1), and reduced the production of NO as low as 10 μM in LPS-induced RAW264.7 cells [154].

Biosynthetic metabolization of alternariol and its 9-*O*-methyl ether is predominantly started with a hydroxylation in 4-position (c.f., chapter on biosynthesis, vide infra), which leads to a variety of further metabolites. 4-Hydroxyalternariol (**19**) was first identified as intermediate of the human, rat, and porcine metabolism [102–103,117,150], but was later similarly found to be a fungal natural product in *Alternaria* sp. [155], *A. tenuissima* [156], and *Trichoderma* (*Hypocrea*) sp. [157]. It is quite astonishing that this assumed main intermediate in the biosynthetic downstream of alternariol was identified as natural product only recently (2021) and that no biological properties were established.

The respective 4-hydroxy derivative of 9-*O*-methylalternariol (**20**) was again initially identified as a product of metabolization [102–103,150], but was similarly found to be a natural product in *Alternaria alternata* [158–161], in further *A.* spp. [147,162–163], and in *Nigrospora* and *Phialophora* sp. [162]. It showed various biological activities: **20** turned out to be antibacterially active, inter alia, against *Pseudomonas syringae* pv. *lachrymans*, *Staphylococcus haemolyticus*, and *Xanthomonas vesicatoria* (IC_50_ values of 7.3, 10.8, and 10.1 µM, respectively) [163], *X. oryzae* pv. *oryzae*, *X. o.* pv. *oryzicola*, and *Ralstonia solanacearum* (MIC: 13.8, 1.7, and 1.7 μM, respectively) [160], and further bacteria [164] and showed antimicrobial activity against *Trypanosoma brucei rhodesiense*, *Leishmania donovani*, and *Plasmodium falciparum* (IC_50_: 13.6, 7.5, 28.3 µM) [159]. It was cytotoxic against soybean cell cultures with an EC_50_ value of 0.63 µM [158] and against cell line 293 (IC_50_: 15.5 µM) [164], showed hydroxyl radical scavenging activity (EC_50_: 68.3 µM) [163], and turned out to be active against 24 tested protein kinases with IC_50_ values ranging from 0.35 to 5.7 µg/mL [147].

Graphislactones A and B (**21** and **22**) were first isolated from the lichens *Graphis scripta* var. *pulverulenta*, *G. prunicola*, and *G. cognata* [43,45–46,165]. Their structures were proposed based on NMR-spectroscopic investigations and were unambiguously confirmed by total syntheses [44,166]. Graphislactone A had already been obtained previously from the synthetic reduction of botrallin (**77**; vide infra) [36–37]. While no further report on graphislactone B was published, graphislactone A was furthermore isolated from various fungi: from *Microsphaeropsis olivacea* [167], *Cephalosporium acremonium* [168], and a further *C.* sp. [169], from *Coniothyrium* sp. [170], from *Rhizopycnis vagum* [171], *Hyalodendriella* sp. [172], *Paraconiothyrium sporulosum* [173], *Pestalotiopsis uvicola* [174], and from *Talaromyces amestolkiae* [175]. Graphislactone A showed moderate activity against acetylcholinesterase (AChE) with an IC_50_ value of 8.1 μg/mL [167] and is a scavenger for the 2,2-diphenyl-1-picrylhydrazyl radical (DPPH; IC_50_: 9.6 μM) and hydroxyl radicals (scavenging activity of 70% and 91% at 0.05 and 0.27 μg/mL, respectively) [169,176]. Furthermore, it turned out to be cytotoxic against SW1116 cells (IC_50_: 9.5 μg/mL) [168].

The trimethyl ether of 4-hydroxyalternariol was named ‘graphislactone H’ (**23**). This might suggest a close relationship to the graphislactones A–F (partly discussed in later sections), which is quite misleading, since **23** is not obtained from the genus *Graphis* and is not a lichen metabolite. It was isolated from *Cephalosporium acremonium* [168], and its structure was determined by NMR spectroscopy and unambiguously confirmed by total syntheses [44,177]. It showed cytotoxic activity against SW1116 cells with an IC_50_ value of 12 μg/mL [168].

**Figure 9:**
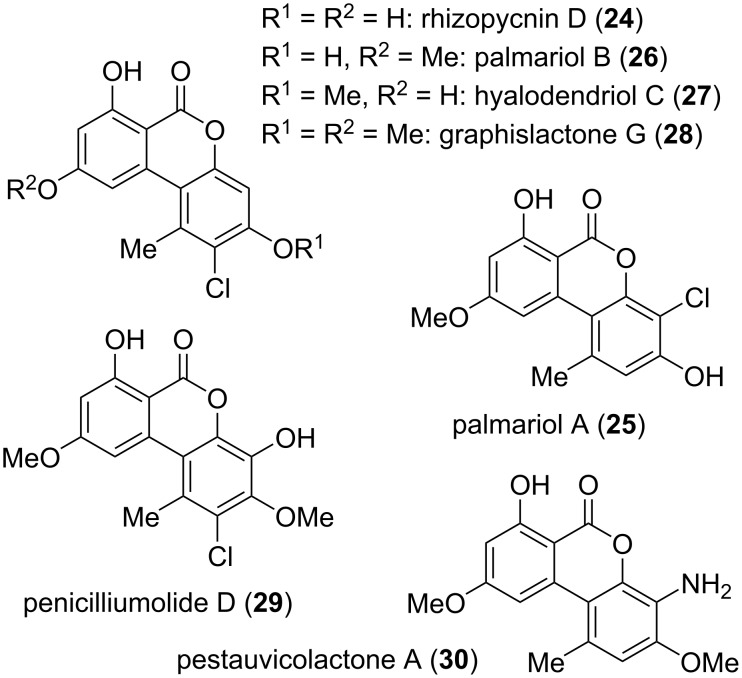
Chloro- and amino-substituted alternariol and its *O*-methyl ethers.

Chlorinated alternariol derivatives (Figure 9) together with the respective redox-modified natural products (Figure 20, vide infra) are a quite wide subclass within the resorcylic lactones, although most of them are observed only sporadic in the organisms. Rhizopycnin D (**24**), i.e., 2-chlorinated alternariol, was isolated from *Rhizopycnis vagum* [171–172]; it inhibited the spore germination of *Magnaporthe oryzae* with an IC_50_ value of 9.9 μg/mL [171].

The 4- and 2-chlorinated derivatives of 9-*O*-methylalternariol, palmariols A and B (**25** and **26**), were first isolated from *Lachnum palmae* [178] and their proposed structures were later unambiguously confirmed by total syntheses [179]. Palmariol B was further found in *Hyalodendriella* sp. [172,180] and *Rhizopycnis vagum* [171]. Palmariol A and B showed weak antifungal activity against *Mucor racemosus* (8 mm inhibition zone at 20 μM/disc) and palmariol A was furthermore active against *Bacillus subtilis* (9 mm) [178]. Palmariol B was found to show a higher antimicrobial activity than 9-*O*-methylalternariol, obviously due to the additional chlorine substituent. It was active against *Agrobacterium tumefaciens*, *Bacillus subtilis*, *Pseudomonas lachrymans*, *Ralstonia solanacearum*, and *Xanthomonas vesicatoria* (IC_50_ values from 16.7 to 18.1 μg/mL) and disclosed antinematodal activity against *Caenorhabditis elegans* (IC_50_: 56.2 μg/mL) [180].

Hyalodendriol C (**27**) was isolated from *Hyalodendriella* sp. [172] and the proposed structure could be confirmed by total synthesis [181]. It proved to be moderately active against *Bacillus subtilis* and *Xanthomonas vesicatoria* with MIC values of 50 and 25 mM, respectively, and showed some larvicidal activity against *Aedes aegypti*.

The 2-chlorinated dimethylated alternariol derivative, graphislactone G (**28**), was (similar as graphislactone H) not isolated from the lichen genus *Graphis* but was obtained from the fungus *Cephalosporium acremonium* [168]. Its structure was confirmed by total syntheses [179,182–183]. Graphislactone G showed a significant cytotoxic activity against SW1116 cells with an IC_50_ value of 21 μg/mL [168].

Penicilliumolide D (**29**), the 2-chlorinated derivative of graphislactone A (vide supra), was first isolated from *Penicillium chermesinum* [184] and later additionally from *Rhizopycnis vagu* [171] and *Hyalodendriella* sp. [172]. It had already previously been obtained as intermediate in a TMC-264 total synthesis [185]. Penicilliumolide D showed some antibacterial activity, was cytotoxic against A549 and HTC116 cell lines with IC_50_ values of 25.5 and 37.3 μM, respectively [171], and exhibited significant larvicidal activity against *Aedes aegypti* (LC_50_: 7.2 μg/mL) [172].

Pestauvicolactone A (**30**) bearing an amino group in position C-4 of dimethylated alternariol was isolated from *Pestalotiopsis uvicola* [174]. It was tested for cytotoxicity, but turned out to be inactive.

**Figure 10:**
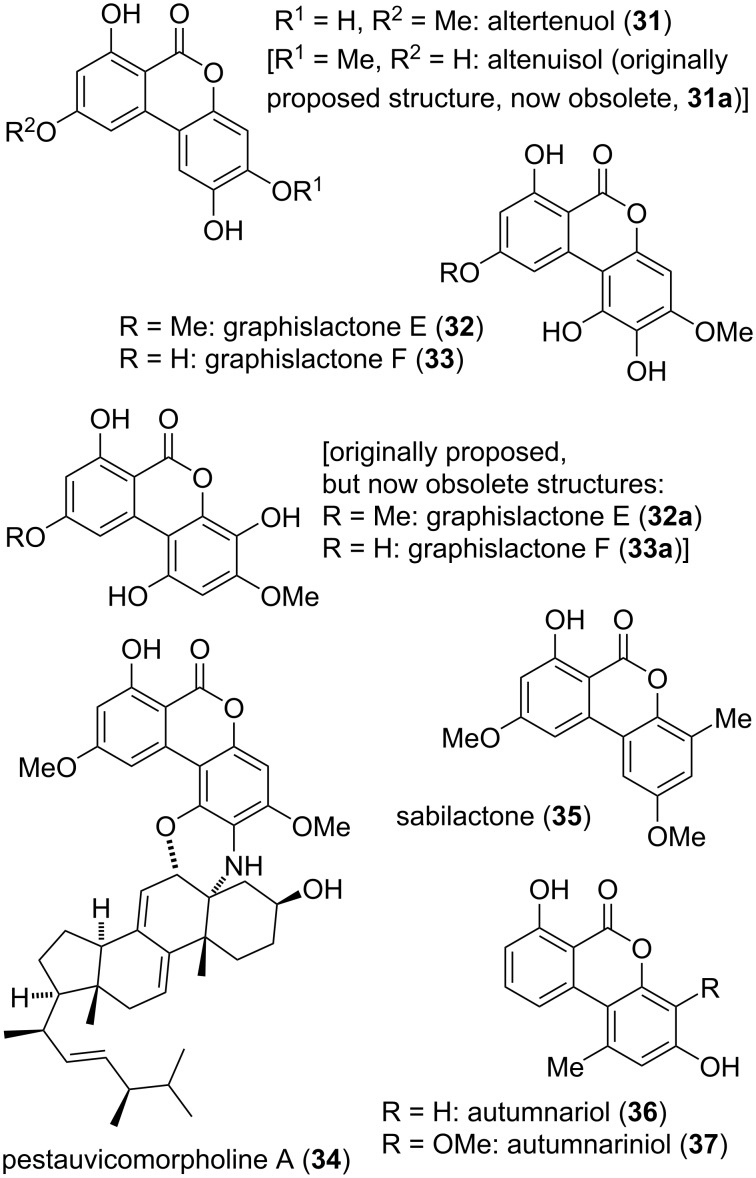
Presumed alternariol derivatives with non-canonical substitution pattern.

Altertenuol (**31**) has already been mentioned in one of the first reports on metabolites isolated from *Alternaria tenuis* (synonymous to *A. alternata*) [42,54]. Its structure was determined by chemical methods and has later been confirmed after total synthesis [186] and comparison of the NMR spectra of synthesized material and of an original sample which survived from the 1950ies (Figure 10) [41]. This study further confirmed that a natural product altenuisol, whose structure **31a** was proposed based on ^1^H NMR-spectroscopic methods, had been assigned incorrectly and its structure is in fact identical with that of altertenuol. The name ‘altenuisol’ and the structure originally proposed with this name are thus obsolete, as is the name ‘verrulactone D’, which is used in the SciFinder database for compound **31**. The latter error is obviously due to a misinterpretation of a scheme in the initial report on verrulactone D [187], a compound not covered in this review. Consequently, all reports on altenuisol are here summarized together with those on altertenuol and the latter name is used throughout. Nevertheless, it has to be realized that the name altenuisol is still used in the community. Besides from *Alternaria alternata*, *A. tenuissima* [188], and various further *Alternaria* sp., altertenuol has been found in *Cladosporium cladosporioides* and *C. sphaerospermum* [189] and has furthermore been isolated as sulfoconjugates [190]. Further total syntheses have been published, partially without noticing the identity with the natural product [191–192]. Altertenuol showed various biological activities, where its toxicity against HeLa cells has already been noted very early (ID_50_ value of 8 μg/mL) together with a toxicity towards *Bacillus mycoides* (even at 5 μg/disc) [40,74]. It was furthermore cytotoxic against A549, MG-63, and SMMC-7721 cell lines (IC_50_ values of 1.47, 2.11, and 7.34 μg/mL, respectively) [123]. It showed antibacterial activity against *Staphylococcus aureus* including methicillin-resistant *S. aureus* (MRSA) and quinolone-resistant *S. aureus* (QRSA), *Bacillus cereus* (MIC values of 8–32 μg/mL [187,193], and further bacteria [163]. It turned out to be active against *Trypanosoma brucei rhodesiense* and *Leishmania donovani* (IC_50_ values of 1.5, 7.1 μM, respectively) and further protozoa [159]. It furthermore showed some radical scavenging activities [163] and phytotoxic effects [194].

Graphislactone E (**32**) was first isolated from the lichens *Graphis scripta* and *G. prunicola* [43] and later similarly from the fungus *Rhizopycnis vagum* [171], while graphislactone F (**33**) was only found in *G. pruniocola* [43]. Their structures **32a** and **33a** were proposed based on NMR-spectroscopic investigations but turned out to be wrong, when the spectroscopic data were compared with those of synthesized material. Re-evaluation of the NMR data and total synthesis of assumed correct structures revealed revised constitutions, which are given in Figure 10 [44]. No biological activity was determined for these compounds.

Pestauvicomorpholine A (**34**) is an exceptional hybrid metabolite with fusion of a polyketide-derived resorcylic lactone and the steroid dihydroergosterol, which was isolated from the fungus *Pestalotiopsis uvicola* [174]. Its structure was determined by NMR spectroscopy; no biological activity could be elucidated.

Sabilactone (**35**) was isolated from the plant *Sabina vulgaris* Antoine [195–196], but no further information could be revealed for this compound. The strong resemblance of autumnariol (**36**) and autumnariniol (**37**) with alternariol and 4-hydroxyalternariol prompted the author to include these compounds into this review although they are not resorcylic lactones. On the contrary, there admittedly is some evidence that these compounds are not derived from alternariol: They were first isolated from the liliaceous plant *Eucomis autumnalis* Graeb [197] and further *E.* sp. [198] (and thus not from fungal sources), they were not isolated together with alternariol or any alternariol derivative, and typical alternariol-derived polyketides seem not to be produced by *Eucomis* sp. [199–201]. Their proposed structures were unambiguously confirmed by total syntheses [202–204], but biological activities were not investigated to date.

**Figure 11:**
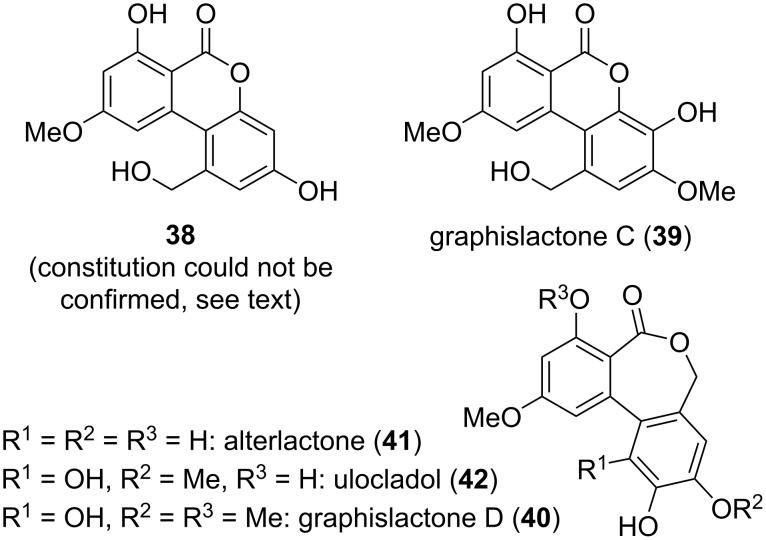
Alternariol derivatives with the 1-methyl group hydroxylated.

Derivatives of alternariol frequently contain additional hydroxy groups attached to the aromatic rings, but there furthermore exist some derivatives, which contain a hydroxylated methyl group. This gives rise to hydroxymethyl-substituted ring systems and opens the possibility of an alternative lactonization with formation of seven-membered lactone rings (Figure 11). Hydroxymethyl-substituted resorcylic lactone **38** was first found to be a product of alternariol metabolization in human, rat, and porcine livers [102], but it was later isolated as natural product from *Trichoderma* sp. [205], *Alternaria alternata* [206–207], and *Pidoplitchkoviella terricola* [208]. Its structure was proposed based on NMR spectroscopy [205], but cannot be confirmed after an unpublished total synthesis, which was finished in the group of this review’s author [209]. The spectra of the synthesized and the natural product did not agree (see Supporting Information File 1). Unfortunately, a corrected structure cannot yet be proposed. Nevertheless, the isolated compound showed some antibacterial activity against *Bacillus subtilis* and *Staphylococcus aureus* (MIC: 64 µg/mL) and DPPH radical-scavenging activity (IC_50_: 12 μg/mL) [205]. It furthermore inhibited *α*-glucosidase with an IC_50_ value of 6.3 µM [206].

Graphislactones C and D (**39** and **40**) were isolated from the lichen *Graphis scripta* var. *pulverulenta* [45,165], graphislactone C was later additionally isolated from *G. prunicola* and *G. cognata*, and graphislactone D was obtained from *G. cognata* [43,46]. Their structures were elucidated by NMR-spectroscopic methods and unambiguously confirmed by total syntheses [44,166]. No biological activities were tested for these compounds.

Alterlactone (**41**) is structurally related with graphislactone D, where two methoxy groups are demethylated to hydroxy groups. It was first isolated from *Alternaria* sp. [147] and later similarly from *Ulocladium* sp. [210], *A. alternata* [159,211–213], and further *A.* sp. [214–216]. The proposed structure could be confirmed by total syntheses [217–218]. Alterlactone showed antimicrobial activity against *Bacillus subtilis* (IC_50_ value of 41 µM) [210], *Candida albicans*, *Trichophyton rubrum* (MIC_80_: 17, 36 µg/mL) [211], *Staphylococcus aureus* (MIC: 31 µg/mL) [214], *Trypanosoma brucei rhodesiense* and *Leishmania donovani* (IC_50_: 7.1, 11.7 µM) [159], and against *Xanthomonas oryzae* pv. *oryzae* (MIC: 16 μg/mL) [212]. It turned out to be a scavenger of DPPH (IC_50_: 99 µM) [210] and showed neuroprotective effects against oxidative injuries [213].

Ulocladol (**42**), a further natural product bearing a seven-membered ring was isolated from *Ulocladium botrytis* [219] and *Microsphaeropsis olivacea* [167]. Its structure was unambiguously confirmed by NMR spectroscopy and by total syntheses [44,220]. Ulocladol is a tyrosine kinase (p56^lck^) inhibitor leading to a reduction of enzyme activity to 7% at 0.02 µg/µL) [219].

**Figure 12:**
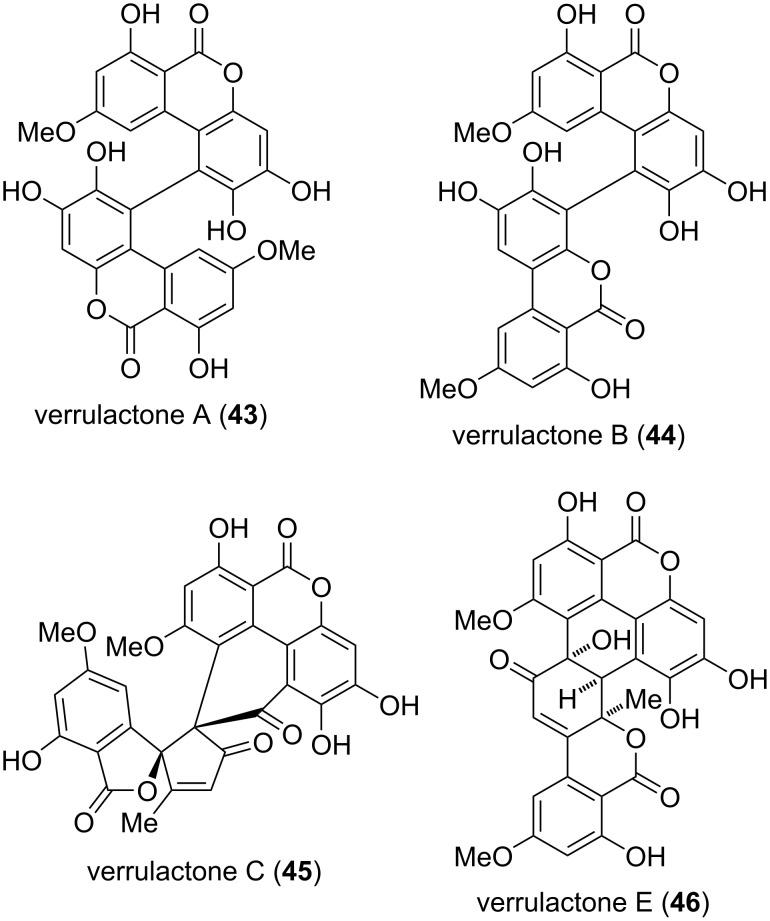
Verrulactones: *pseudo*-dimeric derivatives of altertenuol and related compounds.

Verrulactones A–C and E (**43**–**46**) are dimeric structures, where verrulactones A and B can be considered as altertenuol (**31**) dimers, verrulactone C consists of an altertenuol and an alternaone A (**123**, vide infra) moiety, and verrulactone E contains an altertenuol and a dehydroaltenusin (**74**, vide infra) building block (Figure 12). The verrulactones were isolated from *Penicillium verruculosum* [187,193,221] and verrulactone A and B were further isolated from *Alternaria alternata* [159–160]. Constitution and relative configuration of these compounds was determined by NMR-spectroscopic methods, where the structure of verrulactone A was later unambiguously confirmed by X-ray crystallographic analysis [160]. Chirality and absolute configurations remained unresolved, where it should be noted that even verrulactones A and B are axially chiral with an assumed significant racemization barrier. At least it has been stated, that verrulactone E was not isolated as the racemate since an optical activity was given in the respective report [187]. Verrulactones A–C and E are inhibitors of *Staphylococcus aureus* enoyl-ACP reductase with IC_50_ values of 0.92, 1.41, 16.1, and 24.1 µM, respectively, and verrulactones A and B showed significant antibacterial activity against *S. aureus* including MRSA and against *Bacillus cereus* with MIC values of 8–16 µg/mL. The antibacterial activity of verrulactones C and E was significantly lower (MIC: 32–128 µg/mL) [187,193,221].

#### Biaryls derived from alternariol

**Figure 13:**
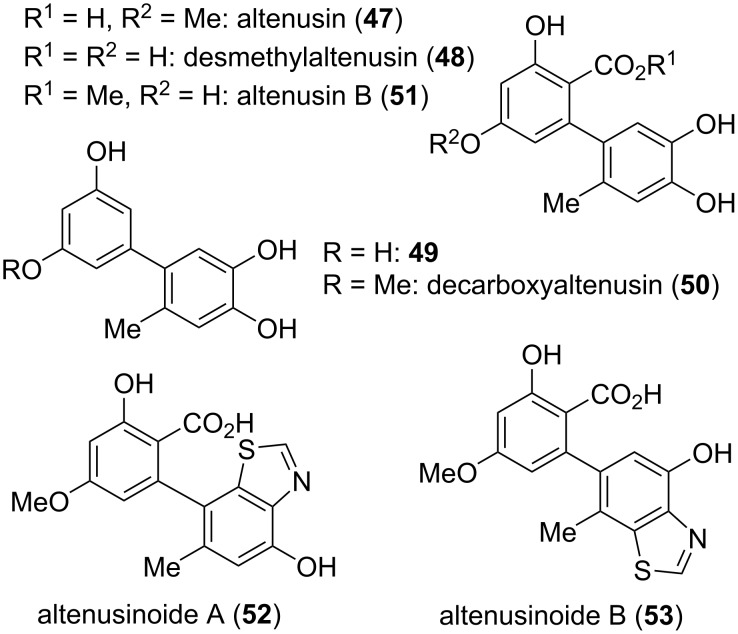
Biaryls formed by reductive lactone opening and/or by decarboxylation.

Although the reductive cleavage of the C_4a_–O_5_ bond in alternariol derivatives leads to biaryls which are thus no longer lactones, the respective derivatives are obviously derived from alternariol and their inclusion in this review seems to be reasonable. Even derivatives downstream in the alternariol biosynthesis, which lack the carboxylic acid are included (Figure 13).

Altenusin (**47**) is biosynthetically obtained through reductive cleavage of 4-hydroxyalternariol (**19**) and it was already mentioned in one of the first reports on Alternaria metabolites when it was isolated from *Alternaria tenuis* (synonymous to *A. alternata*) [42,54]. Its structure was proposed based on NMR-spectroscopic investigations [222] and unambiguously confirmed after total syntheses and comparison of NMR data [217,223–224]. Altenusin was further isolated from *A. longipes* [60] and further non-specified *A.* spp. [147,216,225–226], from *Talaromyces flavus* [49], *T. pinophilus* [227], *Penicillium verruculosum* [228], *P. simplicissimum* [229], and a further unidentified *P.* sp. [228,230], from *Streptomyces verticillus* [228], *Coleophoma* sp. [231], *Ulocladium* sp. [210], *Botryosphaeria dothidea* [232], and from a *Pleosporales* sp. [233]. As one of the most important and highly abundant Alternaria toxins, altenusin was repeatedly investigated with respect of various biological activities. It showed antimicrobial activity against MRSA, *Streptococcus pneumonia*, *Enterococcus faecium*, and *Aspergillus faecalis* (MIC values of 31.3, 31.3, 62.5, 62.5 µg/mL, respectively) [234], against various strains of *Paracoccidioides brasiliensis* (MIC: 1.9–31.2 µg/mL), against *Schizosaccharomyces pombe* (MIC: 62.5 µg/mL) [226], and *Bacillus subtilis* (IC_50_: 39 µM) [210]. It was antifungal against *Botrytis cinereal* (inhibitory efficacy of 56.7% at 200 μg/mL) [235], *Aspergillus fumigatus*, *A. niger*, and *Candida albicans* (zones of inhibition: 16, 15, 12 mm, respectively, at 200 µg/disc) [229], and against *Alternaria alternata* (MIC: 1 µg/mL) [236]. It irreversibly inactivated *Escherichia coli* biotin protein ligase opening a potential application as antimicrobial or biocide [237]. It furthermore turned out to be an inhibitor of trypanothione reductase (IC_50_: 4.3 μM) giving rise to an activity against *Trypanosoma* and *Leishmania* sp. [225] and inhibited *Plasmodium falciparum* dihydroorotate dehydrogenase (PfDHODH) (but not the human orthologue) with an IC_50_ value of 5.9 µM [227]. Altenusin was cytotoxic against Pf3D7 and MRC-5 cells (IC_50_: 60.2, 24.8 µM, respectively) [227], L5178Y cells (IC_50_: 6.8 µg/mL) [147,216], and against the human colorectal HCT 166 cell line (IC_50_: 28.9 µM) [232]. It inhibited 18 different kinases with IC_50_ values ranging from 1.1 to 9.8 µg/mL) [147] and was especially active against the kinase pp60c-Src (IC_50_: 20 nM) [231]. Altenusin turned out to be an inhibitor of α-glucosidase and pancreatic lipase (IC_50_: 46.1, 21.5 µM, respectively) [238], and of neutral sphingomyelinase (nSMase) with an IC_50_ value of 28 µM (but not of aSMase) [230,239]. It inhibited tau aggregation, attracting interest for the treatment of Alzheimer’s disease [240], was a selective agonist of the farnesoid X receptor FXR (EC_50_: 3.2 µM) [241], and displayed remarkable neuroprotective effects against oxidative injuries by acting as potent activator of nuclear factor erythroid-derived 2-like 2 (NRF2) in PC12 cells [213]. Altenusin finally showed radical scavenging activity against DPPH (IC_50_ values in the range of 10.7 and 53 were determined) [210,232,242].

Desmethylaltenusin (**48**), the 9-*O*-demethylated derivative of altenusin has first been obtained from a fungal endophyte *Alternaria* sp. [147] and later from *Talaromyces* sp. [243]. Its structure was elucidated by NMR spectroscopy and later unambiguously confirmed by total synthesis [191]. It was cytotoxic against L5178Y mouse lymphoma cells (EC_50_ = 6.2 µg/mL) [147] and against THLE and HBE cell lines (IC_50_ = 44 and 41 µg/mL, respectively) [243], showed inhibitory potential against various protein kinases with IC_50_ values of 1.5–9.7 µg/mL [147], and displayed significant scavenging activities against nitrite [243].

Biosynthetic decarboxylation of desmethylaltenusin affords biaryl **49**, which was isolated from *Penicillium* sp. [244] and from *Talaromyces* sp. [243]. It showed significant α-glucosidase inhibition with an IC_50_ value of 2.2 μM [244] and was a potent scavenger of DPPH and of nitrite [243].

Decarboxyaltenusin (**50**) was reported in 1974 to be obtained by chemical decarboxylation of altenusin (**47**) and by reduction of dehydroaltenusin (**74**) [37], but was identified as natural product when isolated from *Ulocladium* sp. [210]. It was in the due course additionally obtained from *Nigrospora*, *Alternaria*, and *Phialophora* spp. [162], from further *A.* spp. [242,245], from *A. alternata* [159], *Botryosphaeria dothidea* [232], and from *Pleosporales* sp. [246]. The structure of decarboxyaltenusin was elucidated by chemical [37] and NMR-spectroscopic methods [210] and was later unambiguously confirmed by total synthesis [247]. This compound turned out to be cytotoxic against the human colorectal HCT116 cell line (IC_50_: 73 µM) [232], showed moderate DPPH free radical-scavenging activities (IC_50_: 18.7 µM) [232], exhibited COX-2 inhibitory activity (IC_50_: 9.5 µM) [242], and proved to be antiparasitic against *Trypanosoma brucei rhodesiense* and *Leishmania donovani* (IC_50_ values of 8.3 and 21.5 µM, respectively) [159].

Altenusin B (**51**), the methyl ester of desmethylaltenusin, was isolated from *Alternaria alternata*; it showed neuroprotective effects against oxidative stress-mediated damages in PC12 cells [213].

Two further biaryl derivatives are most likely derived from alternariol and their biosynthesis similarly seems to include a reductive bond cleavage and further transformations. The isomeric altenusinoides A and B (**52** and **53**) were isolated from *Alternaria* sp., where no biological activities were determined so far for these compounds [242].

#### Altenuene and its diastereomers and substituted derivatives

**Figure 14:**
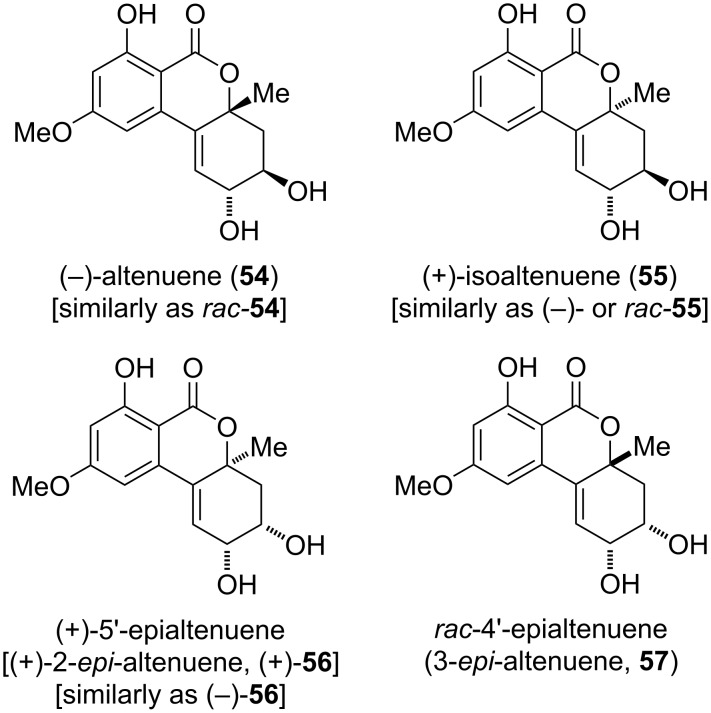
Altenuene and its diastereomers.

**Altenuene and its diastereomers:** Altenuene (**54**) was first isolated from *Alternaria tenuis* (synonymous to *A. alternata*) [38,74,248]. The originally proposed structure turned out to be incorrect (c.f., Figure 3), it was revised after X-ray crystallographic analyses [39,162] of the racemic material and after total synthesis of (−)-altenuene (Figure 14) [249]. While most of the investigations including the X-ray data suggested that this natural product occurs as a racemate [38,74,147,162,248–249], it was occasionally obtained as the (−)-enantiomer [250–251]. The assumption that the methyl group in (−)-altenuene has the same orientation as in isoaltenuene [251] had to be corrected after total synthesis [249] and comparison of measured and calculated ECD spectra [162]. The revised absolute configuration of (−)-altenuene is given in Figure 14. Altenuene was isolated in various *Alternaria* spp., i.e., not only in *A. alternata*, but similarly in *A. capsica-annui*, *A. citri*, *A. porri*, *A. radicina*, *A. tomato* [252], *A. infectoria* [253], *A. tenuissima* [12,252,254], *A. arborescens*, and *A. mali* [12] and it was furthermore found in *Botryosphaeria dothidea* [232], in *Penicillium purpurogenum* [255], and in *Nigrospora* and *Phialophora* sp. [162]. Considering the plethora of reports on this compound (>300 publications), it hardly showed significant biological activities and is less toxic than other Alternaria toxins. [12]. Nevertheless, significant toxic effects of altenuene have been noted as it is acutely toxic to mice (1 of 3 died at 50 mg/kg body weight) [11,74], and showed toxicity against brine shrimp larvae (*Artemia salina*) with an LD_50_ value of 375 µg/mL [256–257]. It exhibited cytotoxicity against HeLa cells (ID_25_: 28 µg/mL) [74], the HCT116 cancer cell line (IC_50_: 3.13 μM) [232], and against MG-63 cells (IC_50_: 17.8 µg/mL) [123]. An antibacterial activity of altenuene was already mentioned in the first report on this compound [38]. It later showed antimicrobial activity against *Staphylococcus aureus*, *Candida albicans* (IC_50_: 39, 13.7 µg/mL, respectively) [138,162,211], and *Bacillus subtilis* (zones of inhibition of 20 mm at 100 µg/disc) [162], and turned out to be a cholesterase inhibitor [258]. The oxidative metabolization of altenuene has been investigated [150,259] and it turned out that it is not metabolized by fecal microbiota [107]. Protocols for its standardized LC–MS/MS analysis have further been developed [121–122].

Isoaltenuene (**55**), the 4a-epimer of altenuene, was first isolated from *Alternaria alternata* [260]. Its proposed structure including its relative configuration was determined by NMR spectroscopy and unambiguously confirmed by total synthesis of the (+)-enantiomer [249]. Whenever the chirality was determined, it was either isolated as (−)-enantiomer (from *A. alternata*) [250], as the (+)-enantiomer (from unidentified freshwater [251] or marine [261] fungi), or as the racemate (from *Nigrospora sphaerica*, *A. alternata*, and *Phialophora* sp.) [162]. It was further isolated without specification of the chirality again from *A. alternata* [138], from *A. brassicae* [262], and further *A.* spp. [215,263], from *Ulocladium* sp. [210], *Colletotrichum capsica* [264], *Setosphaeria* sp. [265], *Phyllosticta capitalensis* [266], and from *Fusarium guttiforme* [267]. Isoaltenuene showed some phytotoxicity on tomato leaves at 20 µg/spot [260], affected seedling growth of amaranth and lettuce [261], and exhibited moderate activity against *Bacillus subtilis* (IC_50_ value of 50.3 µM) [210] and *Staphylococcus aureus* (3.6 mm inhibition zone at 250 µg/mL) [138].

The 2-*epi* diastereomer of altenuene was isolated from *Alternaria alternata* and was given the name ‘5’-epialtenuene’ (**56**) following the numbering in the biphenyl substructure [268]. Although no data on the absolute configuration are given in the initial and in most of the following reports, it turned out that **56** can be found in both enantiomeric forms, where the data are somewhat confusing: Huang et al. claimed that they obtained (+)-**56** from marine fungi, but give a negative specific rotation [261], and Tian et al*.* reported the isolation of (+)-**56** from *Alternaria* sp., but give a specific rotation explicitly only for the 2-*O*-acetyl derivative **63** (vide infra) [163]. Nevertheless, both groups published ECD spectra clearly indicating that the respective isolated compounds are enantiomers. Tian et al. compared the measured ECD spectrum of **63** with a calculated spectrum giving unambiguous evidence that they obtained **63** and thus its deacylated derivative (+)-**56** with the absolute configuration as given in Figure 14 (with a tiny caveat concerning the sign of the specific rotation of **56**). A specific rotation of **56** was furthermore given only once when (+)-**56** was isolated from an unidentified freshwater fungus [251]. 5’-Epialtenuene with non-specified chirality was furthermore isolated from *Penicillium* sp. [269], *Diaporthe* (*Phomopsis*) sp. [152], *Colletotrichum capsica* [264], from *Alternaria alternata* [211], *A. brassicae* [262], and *Pidoplitchkoviella terricola* [208]. **56** (possibly as the laevorotatory enantiomer) showed phytotoxic activity against the germination and growing of amaranth and lettuce and turned out to be antifungal against *Alternaria brassicicola* with an MIC value of 125 µg/mL [261].

A further diastereomer 3-*epi*-altenuene (**57**) was isolated from *Alternaria* sp. and was given the name ‘4’-epialtenuene’ [147]. Its structure including the relative configuration was determined by NMR spectroscopy; the compound turned out to be optically inactive and thus is most likely a racemate. **57** was further isolated from *Trichoderma* sp. [205], *Colletotrichum capsica* [264], again from *Alternaria* spp. [214,216,270–271], from *A. tenuissima* [188], *Pidoplitchkoviella terricola* [208], and from *Fusarium guttiforme* [267]. No biological activities could be determined for this compound.

**Figure 15:**
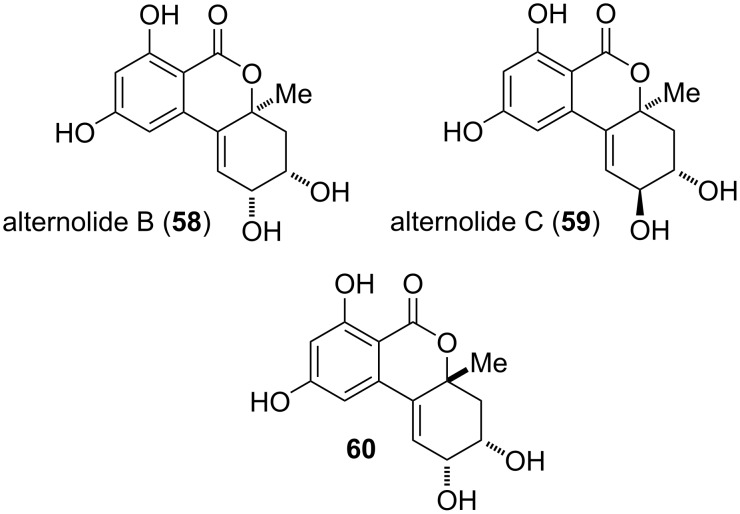
9-*O*-Demethylated altenuene diastereomers.

The natural products depicted in Figure 15 are 9-*O*-demethylated derivatives of altenuene diastereomers. Alternolides B and C (**58** and **59**) were isolated from *Alternaria alternata* and their structures including the absolute configurations were determined by NMR spectroscopy and by comparison of measured and calculated ECD spectra. They showed an insignificant inhibition of *α*-glucosidase with IC_50_ values of 726 and 451 μM, respectively [206]. A further epimer **60** was isolated from *Penicillium* sp. but further data did no become accessible to the author. The given name ‘5-hydroxyaltenuene’ is misleading and its utilization is not recommended [269].

**Figure 16:**
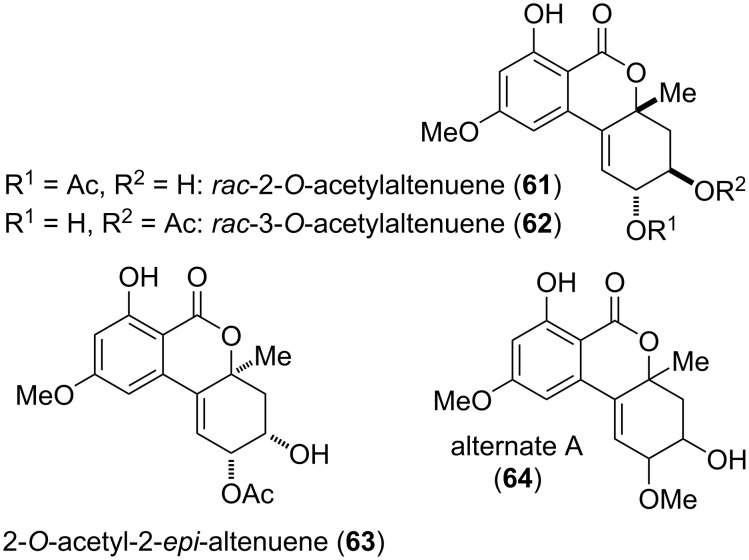
Acetylated and methylated altenuene diastereomers.

**Substituted altenuene and diastereomers:** A number of altenuene diastereomers with further *O*-substituents are given in Figure 16. 2-*O*- and 3-*O*-acetylaltenuene (**61** and **62**) were isolated as the racemates from *Alternaria alternata* [211] and from *Alternaria* sp. [263] and the racemates could be separated by HPLC [211]. 2-*O*-Acetylaltenuene was furthermore obtained from *A. brassicae* [262]. (+)-**61**, (−)-**61**, (+)-**62**, and (−)-**62** showed antimicrobial activity against *Staphylococcus aureus* (MIC_80_: 17, 15, 47, and 45 μg/mL, respectively) and *Candida albicans* (20, 49, 24, >50 μg/mL) [211].

2-*O*-Acetyl-2-*epi*-altenuene (**63**) was isolated from *Alternaria* sp. and its structure including the absolute configuration was elucidated with NMR-spectroscopic methods and by comparison of measured and calculated ECD spectra. No biological activity was determined for this compound [163]. Alternate A (**64**) was isolated from *A. alternata* [161]. The constitution of the compound was elucidated, but neither its absolute nor relative configuration could be determined. Alternate A was tested for its cytotoxic effects but showed no activity.

**Figure 17:**
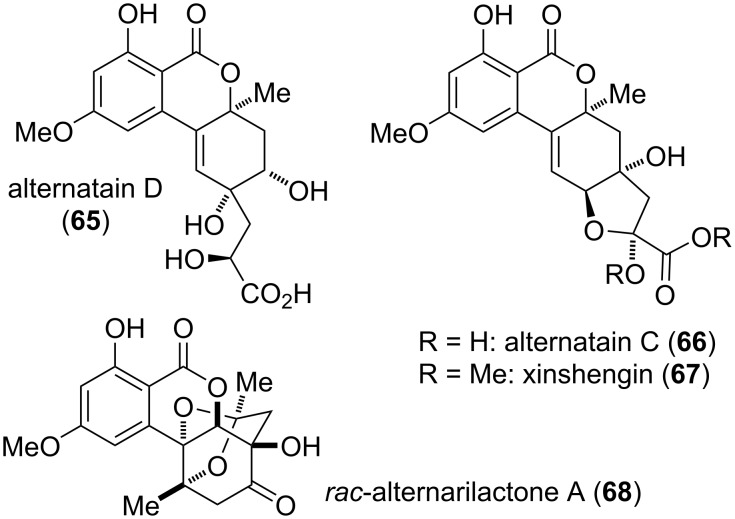
Altenuene diastereomers modified with lactic acid, pyruvic acid, or acetone.

A number of natural products seem to be produced by reaction of altenuene diastereomers (or of related compounds) with C_3_ building blocks, i.e., with lactic acid, pyruvic acid, or with acetone (Figure 17). Alternatain D (**65**) was isolated from *Alternaria alternata* and its structure was determined by NMR spectroscopy and by comparison of measured and calculated ECD spectra. Its structure suggests that dehydroaltenuene B (**86**) might have reacted with lactic acid. Alternatain D inhibited ATP release of thrombin-activated platelets with an IC_50_ value of 58 μM [250]. The biosynthesis of alternatain C (**66**) might similarly involve the addition of pyruvate to dehydroaltenuene B or a related metabolite. It was isolated together with alternatain D but showed no biological activity [250]. Xinshengin (**67**) was independently isolated from *Phialophora* sp. and its structure was again elucidated by NMR spectroscopy and by comparison of measured and calculated ECD spectra. It showed no cytotoxicity against various tested cell lines [272].

Alternarilactone A (**68**) was isolated as the racemate from *Alternaria* sp.; its structure was unambiguously elucidated by X-ray crystallographic analysis showing the addition of an acetone moiety (most likely introduced as acetoacetyl-CoA) to a metabolite similar to penicilliumolide B (**78**) with subsequent acetal formation. Neither of the separated enantiomers showed significant biological activities [273].

**Figure 18:**
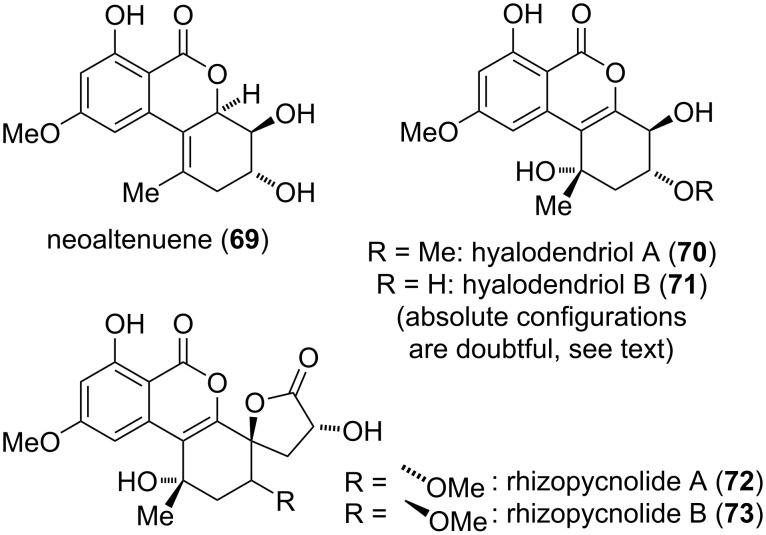
Neoaltenuene and related compounds.

**Neoaltenuene and related compounds:** The compounds in this sub-chapter (Figure 18) have a similar oxidation pattern as altenuene and its diastereomers, but a differing connection and substitution pattern as they bear a methyl group in position C-1 rather than C-4a (as in altenuene). Neoaltenuene (**69**) was first isolated from *Alternaria alternata* [268] and the originally proposed structure was later unambiguously proven by total synthesis [35]. No biological activity has been tested at now for this compound.

The hyalodendriols A and B (**70** and **71**) were isolated from *Hyalodendriella* sp. and their constitutions and relative and absolute configurations were proposed based on NMR-spectroscopic investigations and analysis of the respective Mosher esters [172]. The notorious unreliability of the Mosher method [274] prompted the author to calculate an ECD spectrum [275] of hyalodendriol A giving rise to the assumption that the absolute configuration depicted in Figure 18 might be wrong. This might similarly apply to hyalodendriol B. Details on this calculation are given in Supporting Information File 1.

Hyalodendriol B showed significant activity against larvae of *Aedes aegypti* (LC_50_ value of 20.4 μg/mL) and is an inhibitor of AChE (IC_50_: 21.1 μM) [172].

Rhizopycnolides A and B (**72** and **73**) were isolated from *Rhizopycnis vagum*. Their constitutions and relative and absolute configurations were confirmed by NMR spectroscopy, by comparison of measured and calculated ECD spectra, and by X-ray crystallographic analysis of rhizopycnolide A [171]. Rhizopycnolide A showed moderate antibacterial activity against *Agrobacterium tumefaciens*, *Bacillus subtilis*, and *Pseudomonas lachrymans* (IC_50_ values of 56.7, 45.5, and 44.7 μg/mL, respectively).

#### Oxidized and reduced altenuenes

**Figure 19:**
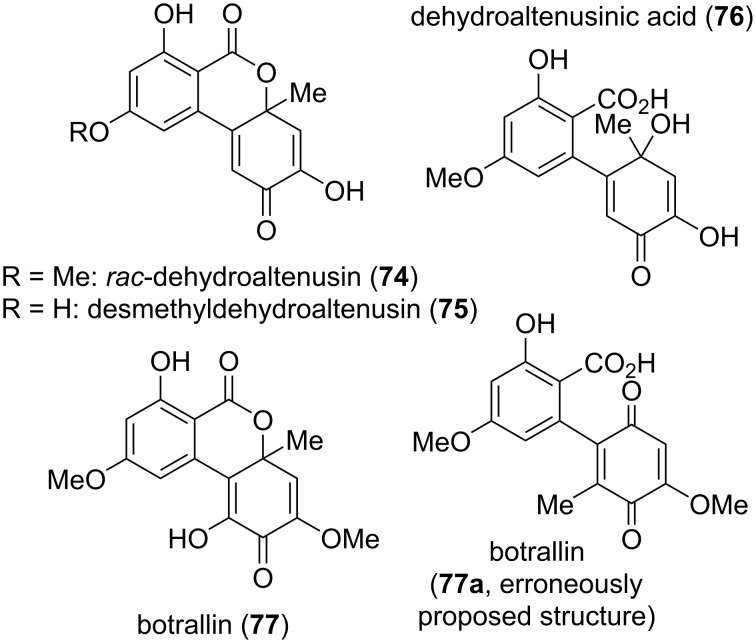
Dehydroaltenusin and its derivatives.

**Dehydroaltenusin and related quinoid compounds:** A number of alternariol derivatives share a quinoid system in the southeastern ring (Figure 19).

**Scheme 1:**
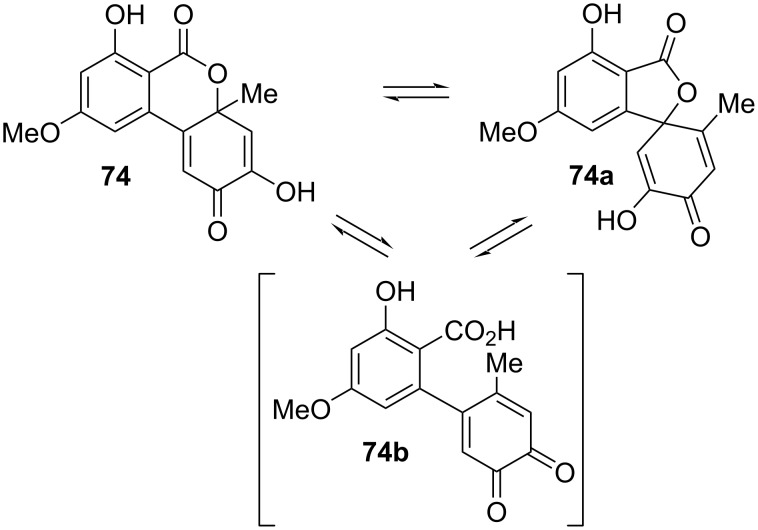
Equilibrium of dehydroaltenusin in polar solvents [278].

Dehydroaltenusin (**74**) was already mentioned 1957 in one of the first reports on Alternaria metabolites by Rosett et al. [54]. Its structure was proposed after evaluation with chemical methods [42,54] and its constitution was later unambiguously confirmed by X-ray crystallographic analysis [276] and by total syntheses [223–224,277]. While the authors of the initial report could not exclude its formation from altenusin by reaction with the charcoal used during the work-up process [42,54], dehydroaltenusin is now commonly considered to be a natural product already present in the filtrates obtained from the organisms. As an optical activity has never been noted for this compound in the first reports and the X-ray crystallographic analysis was performed with racemic crystals [276], one could come to the assumption that dehydroaltenusin is present as racemate in the organisms. Kamisuki et al. reported an equilibrium of **74** with an intermediate ring-opened *ortho*-quinone **74b** and the spiro-fused isomer **74a**, which necessarily leads to a racemization of any enantiopure material (Scheme 1) [278]. While **74** was exclusively present in non-polar solvents like CDCl_3_, the **74a**/**74** ratio is high in polar solvents (D_2_O: **74a**/**74** ratio = 4; DMSO-*d*_6_: 5). This shed new light on the findings of Coombe et al*.* and to a rehabilitation of their defamed work: They proposed structure **74a** for dehydroaltenusin after analysis of NMR spectra measured in the polar solvent acetone-*d*_6_ [222]. The finding that dehydroaltenuene is racemic is of importance for any natural product downstream the biosynthetic path: These might be similarly present as racemates.

Dehydroaltenusin has first been isolated from *Alternaria tenuis* (synonymous to *A. alternata*) [42,54], and later from *Talaromyces flavus* [49,279], *Penicillium verruculosum*, *Streptomyces verticillus* subsp. *tsukushiensis*, and further *P.* sp. [228], from *P. simplicissimum* [229], from a further *S.* sp. [280], from *A. kikuchiana* [281] and *A. angustiovoidea* [282]. It inhibited the cytopathic effects of HIV infection at a concentration range of 1–5 μg/mL, but was cytotoxic to the host cells at 6–8 μg/mL [280]. It furthermore inhibited the calmodulin-dependent activity of myosin light chain kinase (MLCK) with an IC_50_ value of 0.69 μM [228] and had neuroprotective effects against oxidative injuries [213]. It showed antibacterial activity against *Xanthomonas oryzae* pv. *oryzae* and *Bacillus subtilis* with MIC values of 4 [212] and 2 [235] μg/mL, respectively, and a moderate antifungal activity against *Botrytis cinerea* with an IC_50_ value of 11.7 μg/mL [235]. The most important biological activity might be the inhibition of mammalian DNA polymerases (Calf DNA polymerase α, IC_50_: 0.68 mM; the largest subunit of mouse DNA polymerase α, IC_50_: 0.5 μM) [283–285], which was repeatedly investigated in the due course.

Desmethyldehydroaltenusin (also named demethyldehydroaltenusin; [286] **75**) is the 9-*O*-demethylated derivative of dehydroaltenusin (**74**). It has first been isolated from *Talaromyces flavus* [49] and later from *Alternaria* spp. [287]. Its structure was determined by NMR spectroscopy combined with chemical methods [49], where the constitution was later confirmed by total synthesis [191].

Biosynthetic lactone cleavage in dehydroaltenusin (**74**) leads to dehydroaltenusinic acid (**76**), a derivative isolated from *Streptomyces* sp. The authors of the initial report had to admit that they could not completely rule out that this compound might have been formed by hydrolysis of dehydroaltenusin during the isolation and work-up process [288]. However, it showed significant antibacterial activity against nine Gram-positive and -negative bacteria with 21–30 mm zones of inhibition at 100 μg/disk (e.g., against *Staphylococcus aureus*, 23 mm).

Botrallin was already isolated in 1968 from *Botrytis allii* and a quinone-based structure **77a** was originally proposed [36], which was corrected five years later to an isomeric quinoidal structure **77** [37]. Botrallin was similarly obtained from *Microsphaeropsis olivacea* [167] and *Hyalodendriella* sp. [180,289–291]. It showed moderate antimicrobial activity against various bacteria and fungi with IC_50_ values ranging from 82 to 119 μg/mL [180,289–290], was further reported to be active against *Alternaria alternata* with an MIC value of 63 μg/mL [167], and inhibited AChE with an IC_50_ value of 19 μM [167,290].

It seems as if the absolute configurations of desmethyldehydroaltenusin (**75**), dehydroaltenusinic acid (**76**), and of botrallin (**77**) never were elucidated, where an assumed racemic nature of these toxins would go in line with their biosynthetic synthesis from (or parallel with) the racemic dehydroaltenusin (**74**).

**Figure 20:**
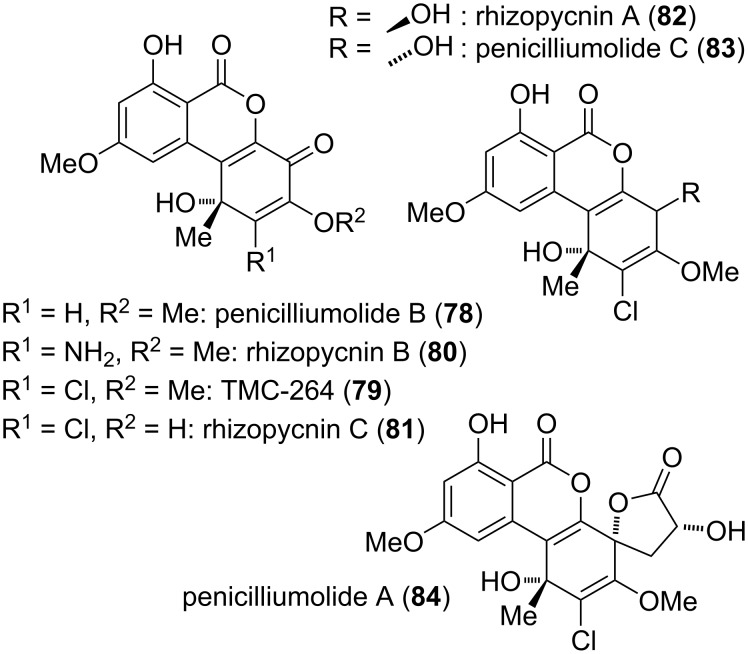
Further quinoid derivatives.

A number of alternariol-derived natural products have a quinonoid structure (i.e., contain a cyclohexa-1,4-diene moiety), but one or two of the quinone carbonyl groups are reduced or otherwise modified (Figure 20). These have been isolated and reported in different batches and thus even closely related compounds got different names. As always in this review, the focus is strictly on structural similarities. Penicilliumolide B (**78**) was isolated from *Penicillium chermesinum*. Its constitution was determined with NMR-spectroscopic methods, but the absolute configuration was only deduced from the compound’s analogy to TMC-264 (**79**) for which a common biosynthetic origin was assumed [184]. **78** was further isolated from *Rhizopycnis vagum* [171] and *Hyalodendriella* sp. [172]. Penicilliumolide B showed cytotoxic activity against the human acute T-lymphoblastic leukemia cell line MOLT-3 (IC_50_ value of 9 μM) but not against eight further cell lines [184]. It turned out to be a moderate AChE inhibitor with an IC_50_ value of 49.6 μM [172].

Rhizopycnin B (**80**), bearing an additional amino group in position C-2, was isolated from *Rhizopycnis vagum* [171], and its absolute configuration was again only deduced from the similarity of its specific rotation with that of TMC-264. No biological activity could be detected for this compound.

TMC-264 (**79**) bearing a chlorine in position C-2 was first detected in *Phoma* sp. [292–293] and later on similarly in *Hyalodendriella* sp. [172,290–291], *Penicillium chermesinum* [184], *Rhizopycnis vagum* [171], and in a marine fungal inoculum [294]. Its structure including its absolute configuration was unambiguously proven by total synthesis, resolution of the synthesized racemate, and X-ray crystallographic analysis of the natural (−)-TMC-264 [185]. TMC-264 showed numerous biological activities, which are most likely due to the quinoid structure and to the presence of the chlorine substituent [293]. TMC-264 suppressed expression of IL-4-driven luciferase and germline Cε mRNA (IC_50_ values of 0.3 and 0.4 μM, respectively) and showed inhibitory activity against tyrosine phosphorylation of STAT5 and STAT6 (IC_50_ values of 16 and 1.6 μM, respectively) [292] and moderately on AChE (IC_50_ value of 79 μg/mL) [172,290]. It was cytotoxic against eight tested cell lines with IC_50_ values ranging from 1.1–12.6 μM (e.g., against HeLA cells: 4.5 μM) [184]. It furthermore turned out to be active against various microorganisms, especially against *Pseudomonas lachrymans* and *Magnaporthe oryzae* with IC_50_ values of 5.9 and 7.5 μg/mL, respectively [171,290], against the nematodes *Bursaphelenchus xylophilus*, *Caenorhabditis elegans*, and *Panagrellus redivivus* (IC_50_ values of 25, 30, 34 μg/mL, respectively) [290], and against the mosquito larvae of *Aedes aegypti* (LC_50_ value of 11.3 μg/mL) [172].

Rhizopycnin C (**81**), the 3-*O*-demethylated derivative of TMC-264, was isolated from *Rhizopycnis vagum* and the absolute configuration was again only deduced from the analogy to TMC-264. It similarly showed significant antibacterial activity, especially against *Pseudomonas lachrymans* and *Staphylococcus hemolyticus* (IC_50_ values of 4.3 and 7.0 μg/mL) [171].

Rhizopycnin A (**82**) and its diastereomer penicilliumolide C (**83**) obviously are biosynthetically derived from TMC-264 by reduction of the quinoid carbonyl group. Rhizopycnin A was isolated from *Rhizopycnis vagum* [171] and penicilliumolide C was obtained from *Penicillium chermesinum* [184]. The constitution of both compounds was determined by NMR spectroscopy and the relative and absolute configuration of **82** was proposed based on measured and calculated ECD spectra [171]. The relative configuration of penicilliumolide C was not published in the initial report [184], but assuming that it is derived from TMC-264 (**79**) and considering the fact that **82** and **83** gave differing NMR spectra (and thus are diastereomers and not enantiomers), the relative configuration of **83** is obviously that given in Figure 20. Nevertheless, although its absolute configuration is very likely as depicted, it was not unambiguously proven. No significant biological activity has been detected for these compounds.

Penicilliumolide A (**84**) is a TMC-264 derivative in which a C_3_-acid (a biosynthetic equivalent of lactic acid) is added to the quinoid carbonyl group establishing a further lactone moiety. It was isolated from *Penicillium chermesinum* [184] and its configuration was proposed based on the quite plausible assumption that its biosynthetic origin again is TMC-264 and on analysis of the Mosher esters. No biological activities were determined for this compound, neither.

**Figure 21:**
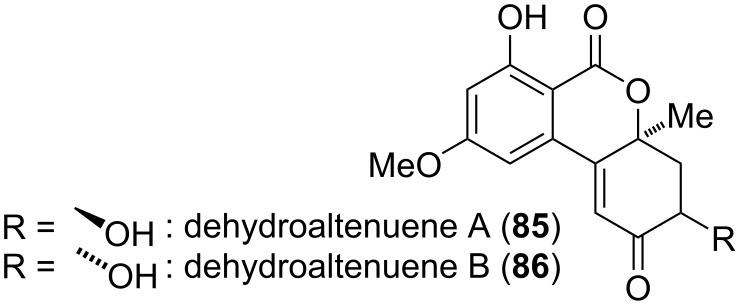
Dehydroaltenuenes.

**Dehydroaltenuene and related compounds:** Dehydroaltenuenes A and B (**85** and **86**, Figure 21) were isolated from an unidentified freshwater fungus [251] and later from *Alternaria brassicae* [262]. Their absolute configuration was proposed based on the analysis of ECD spectra [251] and unambiguously confirmed by total syntheses of dehydroaltenuene A and of *ent*-dehydroaltenuene B [277]. Constitution and relative configuration of dehydroaltenuene B was already proven previously by a total synthesis of the racemic compound [295]. Dehydroaltenuene B turned out to be active against *Staphylococcus aureus* causing a 14 mm zone of inhibition at 100 µg/disk and both dehydroaltenuenes showed activity against *Bacillus subtilis* affording zones of 13 and 20 mm, respectively [251].

**Figure 22:**
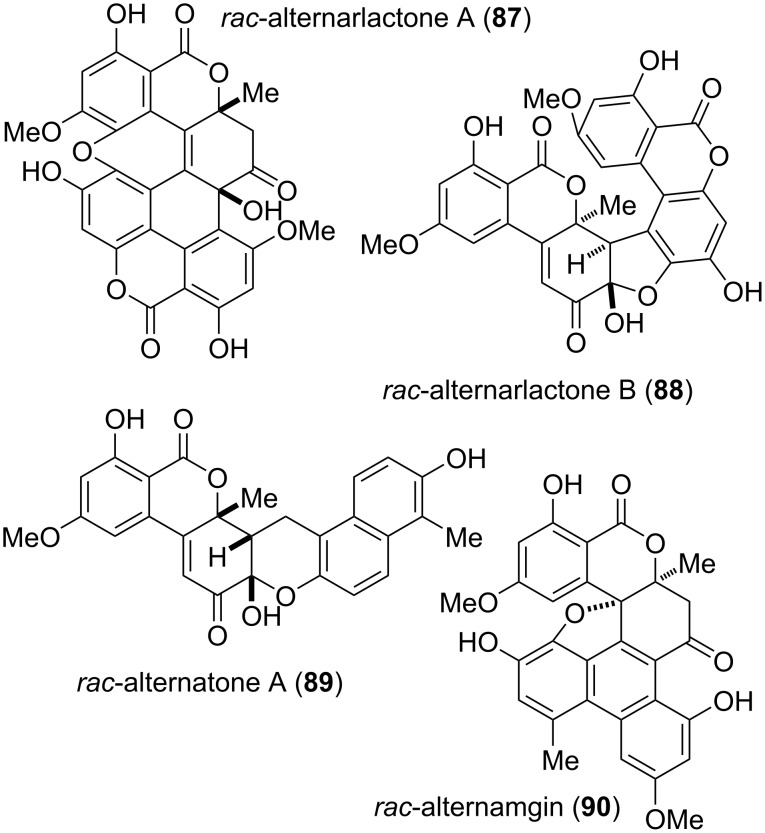
Complex aggregates containing dehydroaltenuene substructures and related compounds.

Some natural products seem to be biosynthetically derived from alternariol, are (at least on a first glimpse) dimeric structures, and contain dehydroaltenuene or related compounds as substructures (Figure 22). Alternarlactones A and B (**87** and **88**) were isolated as racemates from *Alternaria alternata* [159]. Their structures were determined by NMR spectroscopic methods combined with theoretical calculations. They contain two clearly distinguishable alternariol-derived moieties linked in two different modes. Both showed activity especially against *Leishmania donovani* (IC_50_ values of 4.7 and 8.9 μM, respectively) and *Plasmodium falciparum* (5.9 and 9.7 μM).

Alternatone A (**89**) and alternamgin (**90**) contain altenuene substructures, which are augmented by further rings. The former was isolated as a racemate from *Alternaria alternata* and its structure and relative configuration was unambiguously confirmed by X-ray crystallographic analysis [296]. It showed moderate antitumor activity against the human hepatoma carcinoma HepG-2 cell line (IC_50_ value of 38 μM). It should be noted that the name ‘alternatone A’ had already previously been given to a different *Alternaria* metabolite, which is not covered in this review [297]. Alternamgin (**90**) was obtained from *Alternaria* sp. again as the racemate and its structure was similarly elucidated by X-ray crystallographic analysis [298]. After resolution of the enantiomers, these were separately investigated: (−)-**90** showed moderate cytotoxicity against HeLa and HepG2 cells while (+)-**90** was similarly active only against HepG2 cells.

**Figure 23:**
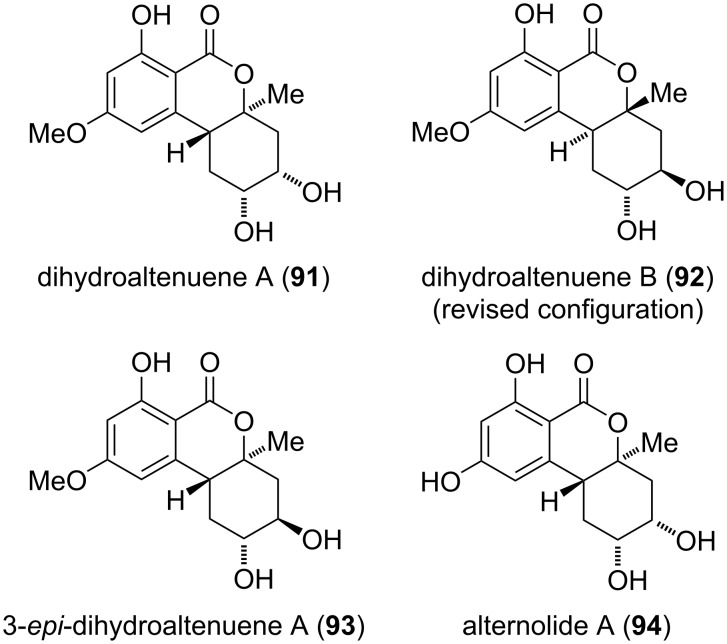
Dihydroaltenuenes.

**Dihydroaltenuene and related compounds:** The dihydroaltenuenes are derived from altenuene and its diastereomers by hydrogenation of the C_1_–C_10b_ double bond (Figure 23). Dihydroaltenuenes A and B (**91** and **92**) have first been isolated in 2006 from an unidentified freshwater fungus [251]. Their constitution and relative configuration was elucidated by NMR spectroscopy [251], but the originally proposed absolute configuration of dihydroaltenuene B (which was deduced only from analogy with biosynthetic precursors) was corrected after total synthesis of the compound [277]. Based on this finding, the given absolute configuration of dihydroaltenuene A might similarly be spurious. Dihydroaltenuene A was furthermore isolated from *Ulocladium* sp. [210], *Alternaria brassicae* [262], and from *Pidoplitchkoviella terricola* [208]; it showed activity against *Staphylococcus aureus*, causing a 14 mm zone of inhibition at 100 µg/disk [251]. Dihydroaltenuene B was similarly found in *Pidoplitchkoviella terricola* [208] and exhibited mushroom tyrosinase inhibitory activity with an IC_50_ value of 38 μM [208].

The diastereomeric 3-*epi*-dihydroaltenuene A (**93**) was first isolated from *Alternaria* sp. and its structure was determined by NMR spectroscopy [163] and by partial synthesis through hydrogenation of isoaltenuene [277]. The absolute configuration was determined by comparison of measured and calculated ECD spectra [163]. It was later furthermore isolated from further *A.* spp. [263,271]. Alternolide A (**94**), the 9-*O*-demethylated derivative of dihydroaltenuene A, was reported only once: It was isolated from *A. alternata* and its structure was confirmed by NMR spectroscopy and by comparison of measured and calculated ECD spectra [206].

#### Altenuic acids and related compounds

**Figure 24:**
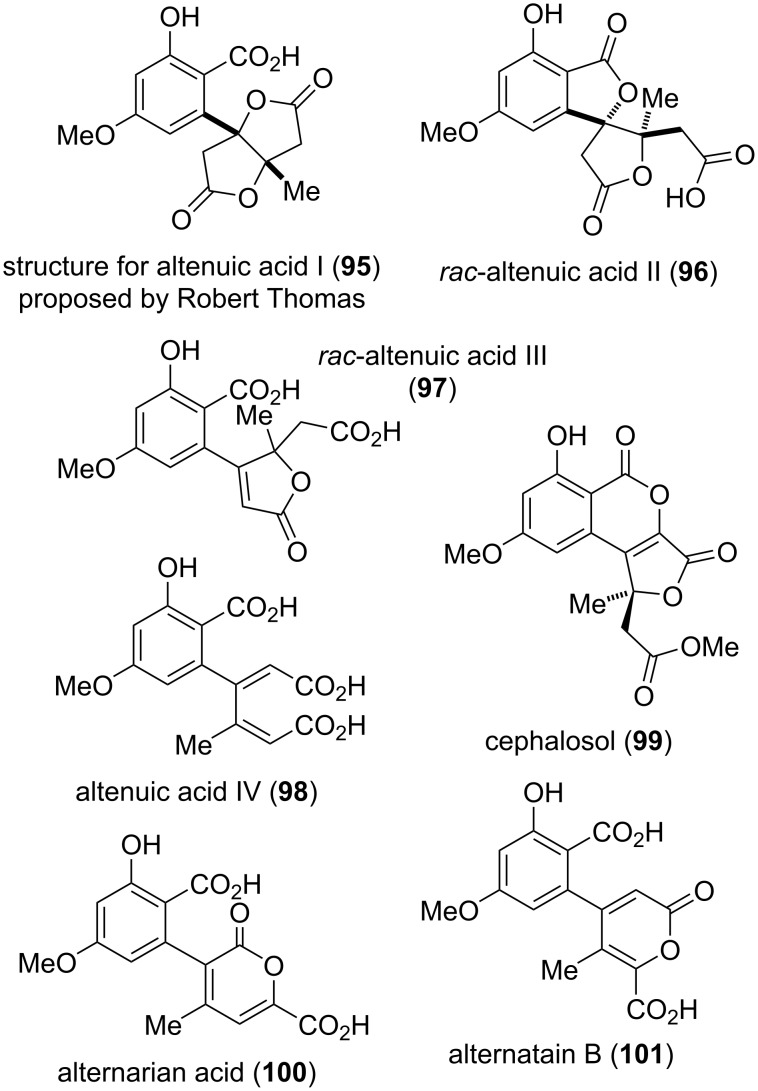
Altenuic acids and related compounds.

The altenuic acids (Figure 24) have first been isolated from *Alternaria tenuis* (synonymous to *A. alternata*) in 1957 by Rosett et al. [54]. Altenuic acids I–III (**95**–**97**) share the same molecular formula (C_15_H_14_O_8_) and show no optical activity. It turned out that both **95** and **96** are easily converted into altenuic acid III (**97**) by dissolving in sodium hydroxide and subsequent re-acidifying. While the structure of altenuic acid II was unambiguously elucidated by X-ray crystallographic analysis [299] and the structure of altenuic acid III was determined by total synthesis of the compound [300], the constitution of altenuic acid I is unclear to date [42]. Nevertheless, the late Robert Thomas, who isolated and investigated altenuic acid I gave a proposal of its structure in a personal communication to the author, which is given in Figure 24. This structure is consistent with all its determined chemical properties, but is unproven [301]. Podlech et al. were given an original sample of a further compound isolated in the 1950ies together with **95**–**97** and they were able to elucidate its structure, which was in the further course confirmed by total synthesis and comparison of the spectra [301]. This toxin was given the name ‘altenuic acid IV’ (**98**); it obviously is a biosynthetic precursor of altenuic acids II and III. None of the altenuic acids showed significant biological activities, where only altenuic acid II was investigated to some extent [159,250,302].

Cephalosol (**99**), a compound closely related to the altenuic acids, has been isolated from the fungus *Cephalosporium acremonium*; its structure including the absolute configuration has been determined [303], where the constitution was unambiguously proven by two independent total syntheses of *rac*-**99** [304–305]. Cephalosol showed significant activities against the human pathogens *Escherichia coli*, *Pseudomonas fluorescens*, *Trichophyton rubrum*, and *Candida albicans* with MIC values of 3.9, 3.9, 7.8, and 2.0 μg/mL, respectively [303].

The isomers alternarian acid (**100**) and alternatain B (**101**) could have similarly been discussed with the biaryl derivatives given in Figure 13 (vide supra). Alternarian acid was first isolated from *Alternaria mali* (synonymous to *A. alternata*) and its structure was confirmed by X-ray crystallographic analysis [306]. It was later again isolated from *A. alternata* [250], from a further *A.* sp. [234], and from *Penicillium* sp. [307]. Alternatain B was obtained from *A. alternata* [250] and from *P.* sp. [307].

#### Cyclopenta-fused derivatives and related compounds

Further degradation of alternariol and its biosynthetic successors can lead to the replacement of the south-eastern (aromatic) six-membered ring with a five-membered ring, where a plethora of hardly related substitution and oxidation patterns were observed. Some of these compounds are recurring metabolites, but most of them were observed only once and have hardly been investigated. In agreement with the organization of the first chapters of this review, these compounds will be treated in subsections according to their structural features and not in first hand considering further parameters.

**Figure 25:**
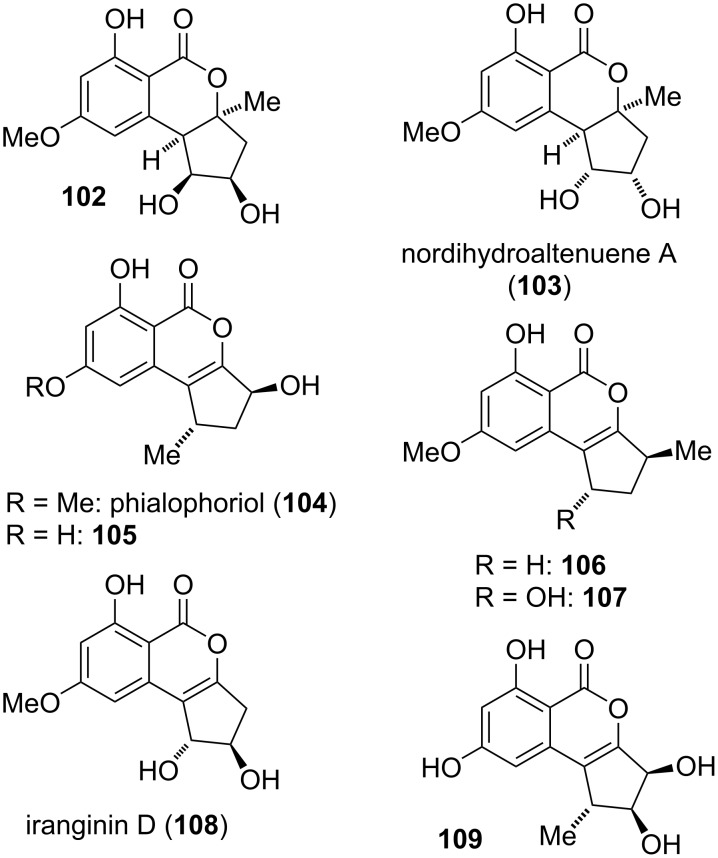
Cyclopentane- and cyclopentene-fused derivatives.

Resorcylic lactone **102** and its diastereomer nordihydroaltenuene A (**103**), bearing a fully saturated cyclopentane ring, were isolated from *Alternaria alternata* [207] and from an *A.* sp. [214], respectively. The structures of both compounds including their absolute configurations were unambiguously confirmed by NMR spectroscopy and X-ray crystallographic analysis (Figure 25) but no biological activity could be revealed for these compounds.

Phialophoriol (**104**) containing a cyclopentene ring has first been isolated in 2013 from an endolichenic fungal strain *Phialophora* sp. [272]. Its structure including relative and absolute configuration was confirmed by NMR spectroscopy and by comparison of measured and calculated ECD spectra. It was furthermore isolated from *Alternaria alternata* [206,211,308], *A. brassica* [262], *Neosartorya glabra* [309], and from *Pidoplitchkoviella terricola* [208]. Some biological activities were tested, but it only showed a moderate activity against *Bacillus subtilis* [211].

The respective metabolite **105** with an unsubstituted hydroxy instead of a methoxy group was isolated from an endophytic *Alternaria* sp. [47]. Its constitution including its absolute configuration was unambiguously confirmed by X-ray crystallographic analysis and by comparison of measured and calculated ECD spectra.

Compounds **106** and **107** have been isolated from an endophytic *Penicillium* sp. [48]. Their structure including the absolute configurations could be determined. Only weak activities against *Staphylococcus aureus* and MRSA were reported. The proposed names ‘deoxytalaroflavone’ (for **106**) and ‘7-hydroxy-deoxytalaroflavone’ (for **107**) are obsolete and should not be used for these compounds. **106** and **107** have no structural relationship with talaroflavone (**122**) and the name ‘deoxytalaroflavone’ had already been assigned to compound **110** in 1990 (vide infra) [49].

Iranginin D (**108**) was isolated from cultures of the ant pathogenic fungus *Ophiocordyceps irangiensis* and its structure was elucidated by X-ray crystallographic analysis [310]. The unnamed compound **109** was obtained from *Alternaria* sp. and constitution and configuration were determined by NMR spectroscopy and comparison of measured and calculated ECD spectra [47]. It showed moderate α-glucosidase inhibitory activity (IC_50_ = 78 µM).

**Figure 26:**
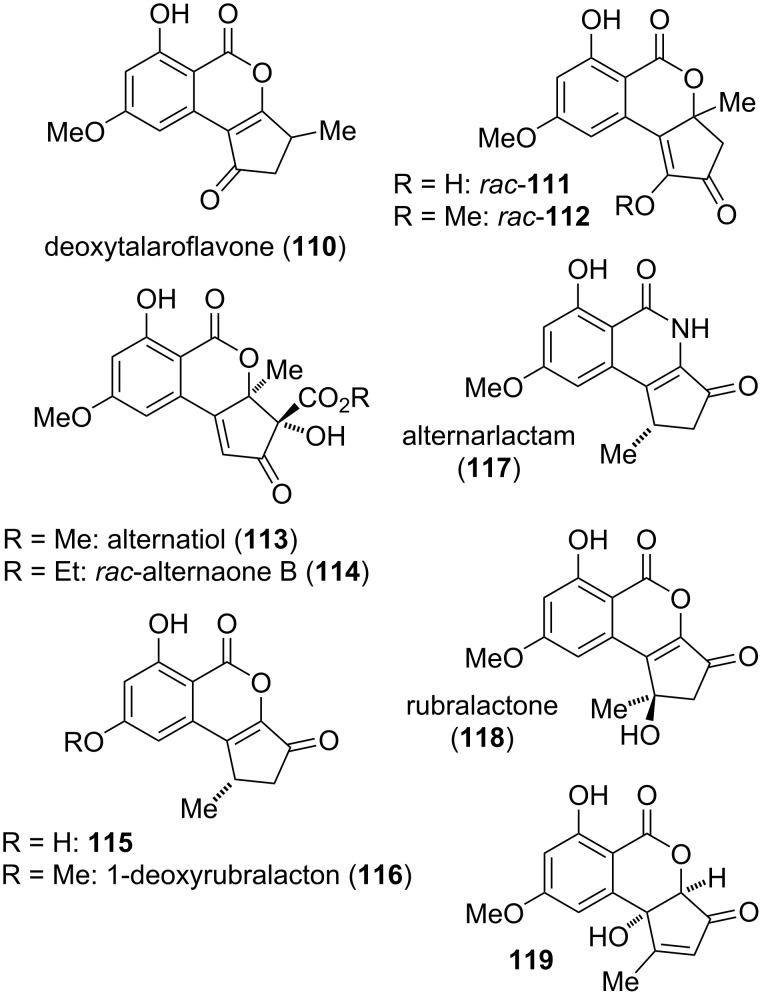
Cyclopentenone-fused derivatives.

Further derivatives contain fused cyclopentenone rings, where the respective enone moieties are located in various positions and orientations (Figure 26). Deoxytalaroflavone (**110**) has been isolated together with talaroflavone (**122**, vide infra) from *Talaromyces flavus* [49]. While the constitution could be proposed based on NMR-spectroscopic analysis, the absolute configuration remained unresolved, due to the small amount of available specimen.

Enone **111** was isolated from endophytic *Alternaria* sp. and its structure was determined by X-ray crystallographic analysis [47]. It showed no optical activity and seems to occur as racemate. Its *O*-methylated derivative **112** was similarly obtained from an *A.* sp.; it was again obtained as racemate [311]. The separated (−)-enantiomer showed only moderate cytotoxicity against the HL-60 (human leukemia) cell line; its (+)-enantiomer turned out to be inactive.

Alternatiol (**113**) was first obtained from *Alternaria alternata* as the enantiomer depicted in Figure 26 [308]. Later on, it was further isolated together with alternatone B (**114**) from a different *A. alternata* strain, where this time both compounds were obtained as racemates. Their structures were unambiguously determined by X-ray crystallographic analysis [235].

Compound **115** was isolated from *Alternaria* sp. and its constitution and absolute configuration were determined by NMR spectroscopy and comparison of measured and calculated ECD spectra [47]. A moderate α-glucosidase activity (IC_50_ value of 78 µM) was determined for this compound.

1-Deoxyrubralactone (**116**) was first isolated from a fungal strain derived from sea algae [312] and later from *Penicillium pinophilum* [313], *Alternaria alternata* [161,206,250], *A. brassicae* [262], and from further *A.* sp. [47,214,314], from *Setosphaeria* sp. [265], *Pidoplitchkoviella terricola* [208], and *Talaromyces pinophilus* [315]. Its constitution was determined by NMR spectroscopy [312] but the absolute configuration could only be elucidated 14 years after the first report by comparison of measured and calculated ECD spectra [206] and later by X-ray crystallographic analysis [315]. 1-Deoxyrubralactone showed inhibition of rat DNA polymerase β and human polymerase κ with IC_50_ values of 11.9 and 59.8 µM, respectively [312], α-glucosidase inhibition (IC_50_: 1.6 µM) [206], and moderate cytotoxic activity against cell lines Huh-7 and A549 with IC_50_ values of 93 and 91 µg/mL, respectively [265].

Due to the close relationship of alternarlactam (**117**) with 1-deoxyrubralactone (**116**), it is discussed in this review, although it obviously is no lactone. It is the only lactam closely related and presumably biosynthetically derived from alternariol, which came to the author’s attention. Alternarlactam was first obtained from an *Alternaria* sp. [316], later similarly from *A. alternata* [250], and from *A. tenuissima* [188]. Its structure including its absolute configuration was elucidated by NMR spectroscopy, comparison of measured and calculated spectra, and by total synthesis of the compound [316]. The natural (−)-enantiomer inhibited the growth of human cervix HeLa adenocarcinoma cells (IC_50_: 1.10 µg/mL) and of the human hepatocellular carcinoma cell line QGY-7701 (IC_50_ = 1.52 µg/mL). The synthesized (presumably unnatural) (+)-enantiomer turned out to be less effective [316]. Alternarlactam furthermore showed an antiplatelet effect (activity on the ATP release of thrombin-activated platelets) with an IC_50_ value of 69 µM [250].

Rubralactone (**118**) was isolated in 2007 from *Penicillium rubrum* and its constitution was proposed based on NMR spectroscopy [317]. It was further isolated from *P. purpurogenum* [255], from an endolichenic fungus *Ulocladium* sp. [210], from *Alternaria* sp. [47,215], and from an entomogenous *Setosphaeria* sp. [265]. Although rubralactone showed optical activity [317] its absolute configuration apparently has never been determined. Nevertheless, one might assume that its configuration goes in line with that of the related 1-deoxyrubralactone (**116**).

Compound **119** was isolated from a *Penicillium* sp. [318]. Its absolute configuration was proposed after analysis of the measured ECD spectrum. Only a moderate root growth inhibition in the germination of *Arabidopsis thaliana* was determined with this compound.

**Figure 27:**
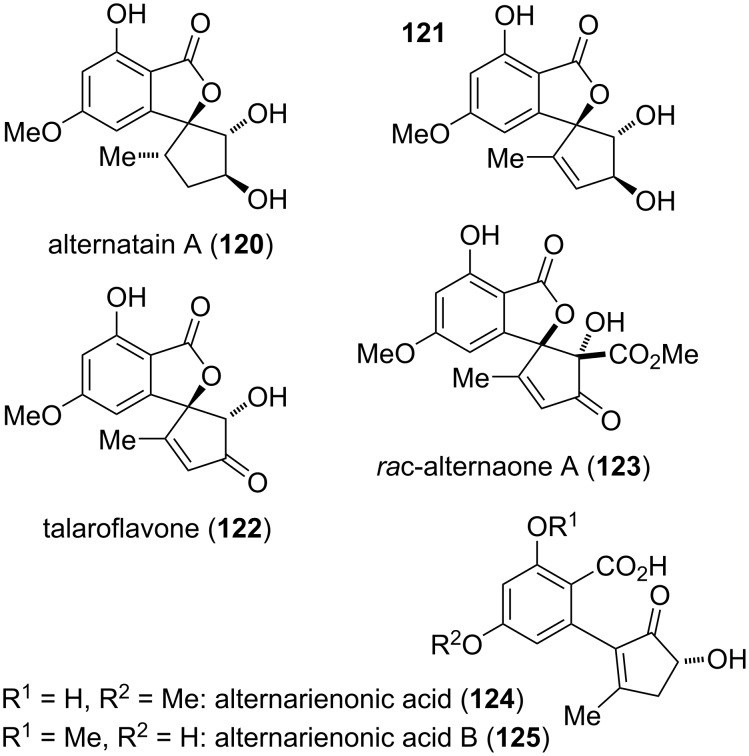
Spiro-fused derivatives and a related ring-opened derivative.

The structural complexity of alternariol-derived natural products is even higher in some spiro-fused compounds (Figure 27). Alternatain A (**120**) was isolated in 2019 together with further (structurally deviating) alternatains (vide supra) from *Alternaria alternata* [250]. Its structure including the relative and absolute configuration was confirmed by NMR-spectroscopic methods and by comparison of measured and calculated ECD spectra. No biological activity was determined for this compound.

The related dehydro compound **121** was isolated from an *Alternaria* sp. [47] and later from *Pidoplitchkoviella terricola* [208]. Its structure was confirmed by NMR spectroscopy and by comparison of measured and calculated ECD spectra [47]. It showed no α-glucosidase or mushroom tyrosinase inhibitory activity [47,208].

Talaroflavone (**122**) was first isolated from *Talaromyces flavus* [49]. Its constitution was assigned based on NMR spectroscopy in the initial report, but its relative and absolute configuration was only proposed 26 years later by a combination of chiroptic methods and DFT calculations for comparison [255]. Talaroflavone was repeatedly isolated from various further sources: from *Alternaria brassicae* [262], and further *A.* sp. [47,147,215–216,238,270], from *Pidoplitchkoviella terricola* [208], *Penicillium purpurogenum* [255], and from further unidentified fungals [251,312]. It showed inhibition of rat DNA polymerase β and human polymerase κ with IC_50_ values of 16.3 and 86.5 µM, respectively [312], and was a moderate inhibitor of pancreatic lipase (IC_50_: 74 µM) [238].

Alternaone A (**123**) was isolated as the racemate from *Alternaria alternata* and its structure was unambiguously determined by X-ray crystallographic analysis [235]. Both enantiomers showed moderate antibacterial inhibition on *Xanthomonas oryzae* pv. *oryzae* and pv. *oryzicola* (MIC values of 100 µg/mL for both pathovars).

The alternarienonic acids (**124** and **125**) are somewhat related to the spiro compounds in Figure 27 and are thus discussed here. Alternarienonic acid (**124**) was first isolated from *Alternaria* sp. [147,234], its structure was determined by NMR-spectroscopic methods, and the absolute configuration was proposed after analysis of the respective Mosher derivatives [147]. However, when **124** was isolated from *A. alternata*, *Nigrospora sphaerica*, or *Phialophora* sp., the data suggest that it was isolated as racemate or possibly as the respective enantiomer [319]. It was later isolated from further *A. alternata* strains [250] and from unidentified *A.* sp. [238,242]. Alternarienonic acid showed significant α-glucosidase inhibition with an IC_50_ value of 8.0 µM and a somewhat lower inhibition of pancreatic lipase (IC_50_: 20.8 µM) [238]. The isomeric alternarienonic acid B (**125**) was isolated from an *Alternaria* sp. [270].

**Figure 28:**
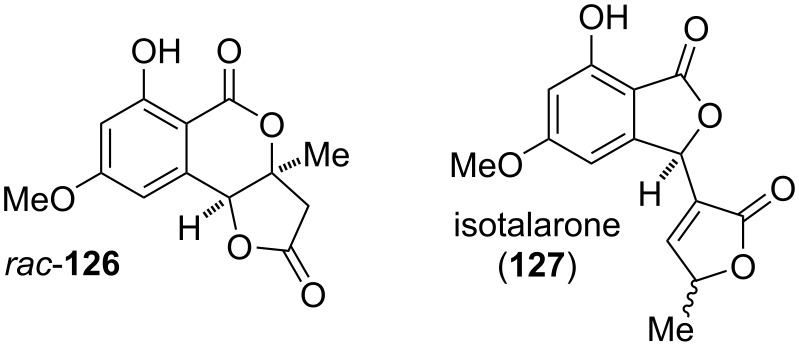
Lactones-fused and lactone-substituted derivatives.

Compound **126** (Figure 28) was first isolated as the racemate from an endolichenic fungus *Ulocladium* sp. [210] and later from *Alternaria brassicae* [262], *A. alternata* [207], and a further *A* sp. [214], from *Pidoplitchkoviella terricola* [208], and from a *Penicillium* sp. [307].

Isotalarone (**127**), isolated from *Penicillium purpurogenum*, is a derivative with even less relationship to its presumed precursor alternariol [255]. Its structure was claimed based on simulated NMR spectra and on calculated ORD data; it seems to be present as an inseparable mixture of diastereomers. It could be noted that a natural product talarone was never reported; the name ‘isotalarone’ was given to indicate its isomerism with talaroflavone (**122**). Isotalarone showed moderate activity against Gram-positive bacteria (*Bacillus subtilis*, inhibition zone Ø 15 mm; *Streptomyces viridochromogenes*, 16 mm) and moderate toxicity against brine shrimps (8%, 10 µg/mL).

### Biosynthesis of alternariol and its derivatives

The biosynthesis of alternariol (**1**) and its very closely related metabolites has been investigated in detail, but hardly any evidence is available for the biosynthesis of further derivatives.

**Scheme 2:**
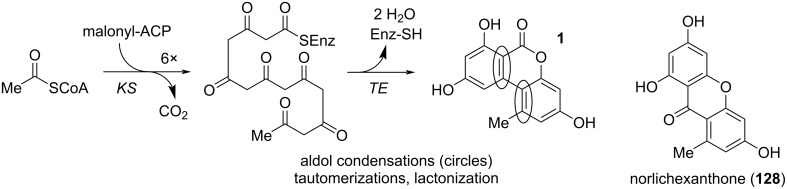
Biosynthesis of alternariol [324].

It has already been elucidated by feeding with 1-[^14^C]-labeled acetic acid that alternariol is built from acetic acid units and the orientation of these in the natural product could be established [58–59,320]. Further investigations used deuterated precursors and came to equivalent conclusions [321]. The finding that alternariol is produced via norlichexanthone (**128**) [322] was refused soon after [323]. Alternariol is a prototypic polyketide (not to say *the* prototype polyketide), which is produced from acetyl-CoA and six malonyl-CoA units by the ketosynthase (KS) moiety of a polyketide synthase (PKS). Liberation from the enzyme by a transesterase (TS) with concomitant lactonization, aldol condensations, and a number of tautomerizations establishing the aromatic rings furnish alternariol without the need for further transformations (Scheme 2) [324].

**Scheme 3:**
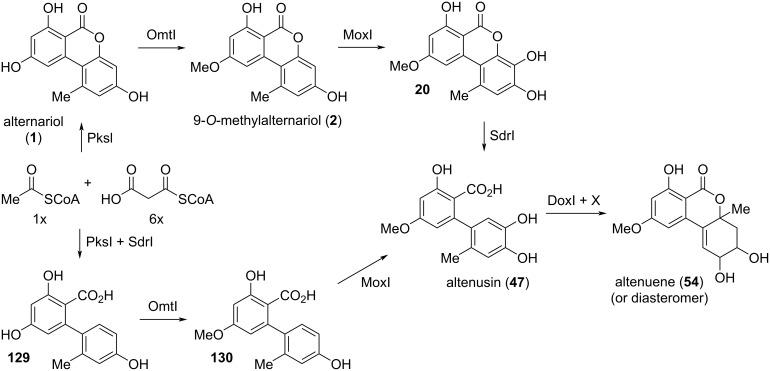
Biosynthesis of alternariol and its immediate successors with the genes involved in the respective reactions [325].

Biosynthetic derivatization of alternariol with formation of the main Alternaria toxins has been thoroughly investigated by Fischer et al., who identified the herein involved genes (Scheme 3) [325]. One path includes the formation of alternariol (**1**) involving a polyketide synthase gene (PksI), an *O*-methyltransferase (OmtI) leads to the formation of 9-*O*-methylalternariol (**2**), an FAD-dependent monooxygenase (MoxI) is responsible for the oxidation to the unnamed metabolite **20**, a short-chain dehydrogenase (SdrI) probably catalyzes the reductive formation of altenusin (**47**), and finally a putative extradiol dioxygenase (DoxI) leads to the formation of altenuene **54**. Nevertheless, the last transformation requires further, yet unknown steps. The authors proposed a possible alternative pathway in which PksI together with SdrI encodes the formation of a reduced biaryl derivative of alternariol **129**, which is further methylated to methyl ether **130** and oxidized to altenusin (**47**).

**Scheme 4:**
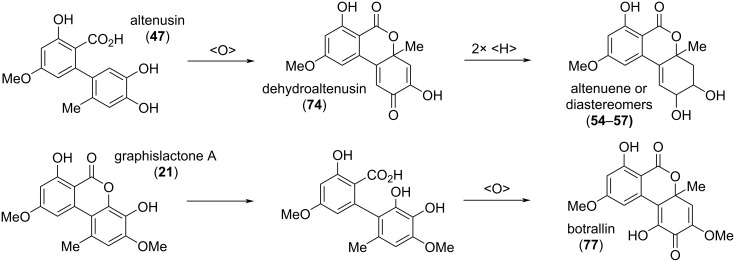
Presumed formation of altenuene and its diastereomers and of botrallin.

Dehydroaltenusin (**74**) is obviously an oxidation product of altenusin (**47**) [49], where it was shown that this oxidation can be achieved enzymatically with horseradish peroxidase (Scheme 4) [281]. Keeping in mind that dehydroaltenusin is racemic (c.f., Scheme 1), its reduction to altenuene (and its diastereomers) gives rise to two further stereogenic centers, and that all the possible enantiomers and diastereomers (in total, eight stereoisomers) have been identified as natural products, one might conclude that reduction of **74** to diastereomers **54**–**57** might be rather unselective. Which of the diastereomers are formed and which enantiomers appear predominantly (if at all) might be ruled to some extend by the respective organism, by further environmental conditions, or by the applied isolation methods.

A biosynthetic path for the formation of botrallin (**77**) seems not to have been proposed yet, but it would be accessible from a 4-hydroxylated alternariol derivative by hydrolysis of the lactone and oxidative lactone/quinone formation. The required methoxy groups could be built at any stage, but graphislactone A (**21**) would be an obvious precursor.

**Scheme 5:**
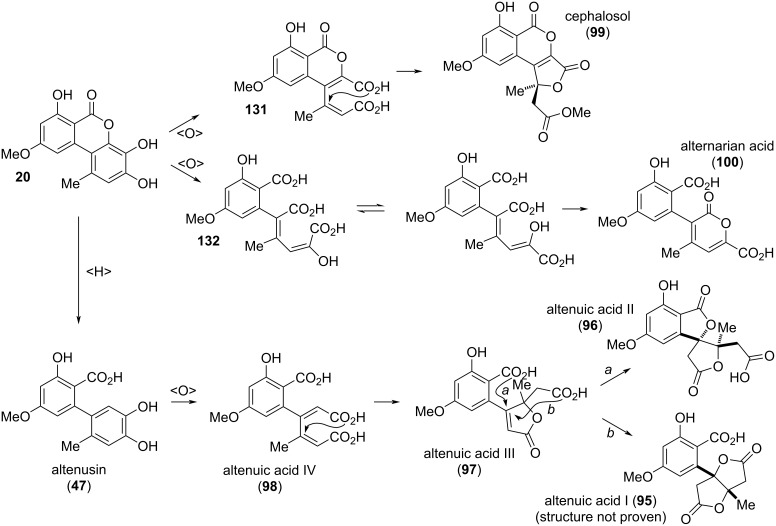
Presumed formation of altenuic acids and related compounds.

There is also hardly any evidence for the biosynthetic processes leading to further metabolites, but plausible proposals have been made for most of them. Scheme 5 gives a selection of metabolic paths as given in the literature, where only significant changes in the metabolites’ structures are depicted. Not all proposals for simpler *O*-methylations, hydroxylations, as well as oxidation and reduction steps are generally included.

The oxidative degradation of catechol substructures is a major pathway in the metabolization of aromatic compounds [326]. Respective oxidations of 4-hydroxylated alternariol derivatives (e.g., **20**) and altenusin (**47**) open up an access to significantly altered metabolites (Scheme 5). Oxidative cleavage of **20** leads to a putative dicarboxylic acid **131**, where conjugate addition at the bottom enoate would lead to cephalosol (**99**) [303]. An equivalent oxidative path would lead to tricarboxylic acid **132**, which after isomerization of the bottom double bonds (possible via keto–enol tautomerizations) and lactonization would yield alternarian acid (**100**) [306]. Oxidative ring-opening of the catechol substructure in altenusin (**47**) would yield altenuic acid IV (**98**), where a first conjugate addition would give rise to altenuic acid III (**97**) and a second addition would furnish either altenuic acid II (**96**) or the speculative structure of altenuic acid I (**95**), depending on the mode of attack [301].

**Scheme 6:**
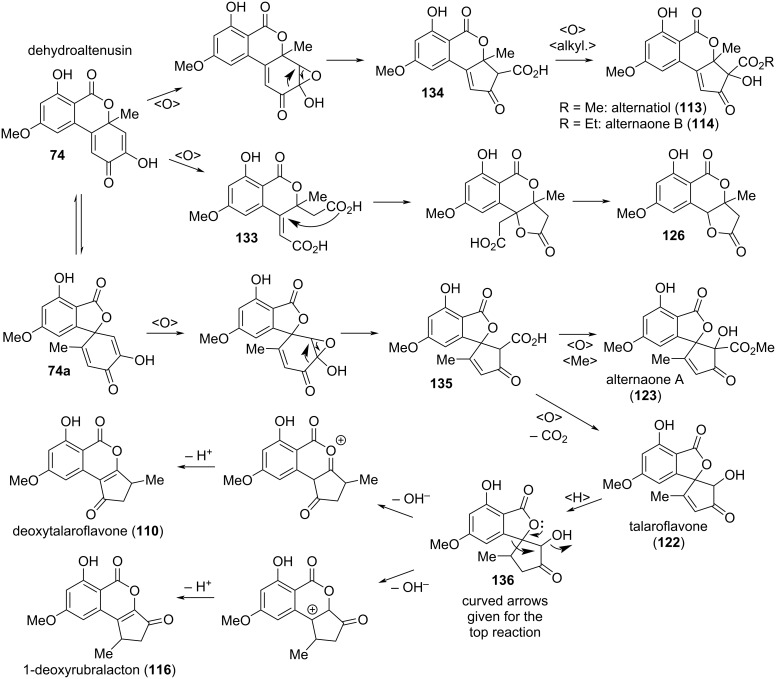
A selection of plausible biosynthetic paths to cyclopenta-fused metabolites. (No stereochemistry is indicated in this scheme.)

The cyclopenta-fused resorcylic lactones show very divers structural patterns in their ring fusion and substitution, where all of them require transformation of the ring system, usually through a rearrangement. All the proposed metabolic paths given in Scheme 6 start with dehydroaltenusin (**74**) as either of the isomers present in its equilibrium (c.f., Scheme 1). Oxidative opening of the quinoid ring in **74** would lead to a dicarboxylic acid **133**; conjugate addition and elimination of acetic acid gives rise to the unnamed compound **126** [210]. Oxidation of **74** to an epoxide could be followed by Meinwald-type rearrangement yielding carboxylic acid **134**, whose alkylation would furnish alternatiol (**113**) and alternaone B (**114**), respectively [235]. If the spiro-fused isomer of dehydroaltenusin **74a** is oxidized to an equivalent epoxide, its rearrangement would yield a carboxylic acid **135**, whose hydroxylation and methylation could lead to alternaone A (**123**) [235]. Although formation of talaroflavone (**122**) seems to have not been proposed in the literature, one could assume, that it might arise from hydroxylation in **135** and subsequent decarboxylation. Reduction of **122** would lead to spiro-fused cyclopentanone **136**, which can be rearranged involving the migration of the aryl group attached to the spiro center, loss of hydroxide, and final deprotonation, furnishing deoxytalaroflavone (**110**) [49]. Although not proposed in the literature, it might similarly come to the migration of the carboxyl group in **136**. A similar mechanistic sequence would thus give rise to 1-deoxyrubralacton (**116**), differing from **110** only in its substitution pattern.

### Total syntheses of alternariol-derived resorcylic lactones

For many of the compounds mentioned in this overview total syntheses have been presented, but only those following significantly differing strategies, will be discussed herein in some detail.

**Scheme 7:**
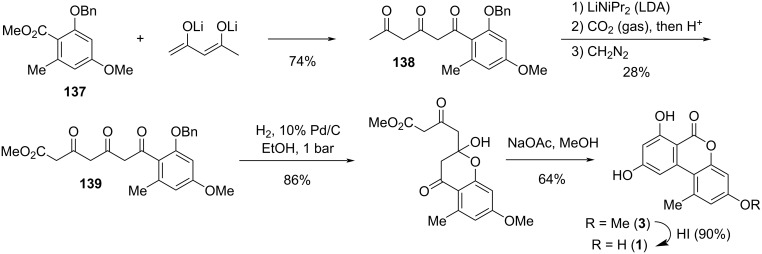
Biomimetic synthesis of alternariol (**1**) by Harris and Hay [66].

The first syntheses in this field followed a biomimetic approach [65–68], in which a polyketide (which is in its very own meaning a long-chain carboxylic acid alternately bearing carbonyl and methylene groups in its backbone) [327] is successively assembled and condensated to the final product. Harris and Hay started with a suitably protected orsellinic acid derivative **137** (accessible from methyl 3,5,7-trioxooctanoate [328]) and reacted it with the dilithium bisenolate of acetylacetone to yield triketone **138** (Scheme 7). Enolate formation with lithium diisopropylamide (LDA), reaction with carbon dioxide, and immediate formation of the methyl ester gave rise to **139**. Debenzylation with hydrogenolytic conditions and condensation/lactonization with mildly basic conditions furnished 3-*O*-methylalternariol (**3**), which was finally subjected to an ether cleavage with hydroiodic acid furnishing alternariol (**1**). A quite similar biomimetic approach has also been used by Langer et al. for the total synthesis of autumnariol (**36**) [204].

**Scheme 8:**
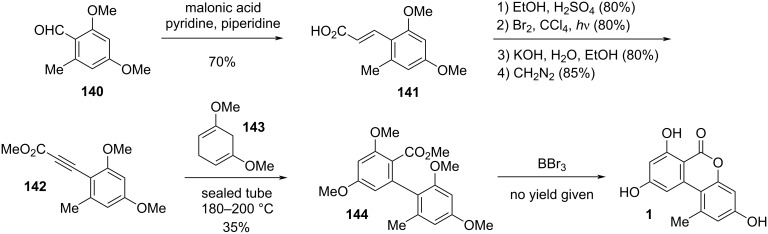
Total synthesis of alternariol (**1**) by Subba Rao et al. using a Diels–Alder approach [34].

An alternative approach presented by Subba Rao et al. started with benzaldehyde **140**, which was transferred into the respective cinnamoic acid **141** by Knoevenagel–Doebner condensation (Scheme 8). The double bond was dibrominated and eliminated to methyl propiolate **142**. Reaction with 1,4-cyclohexadiene **143** in a sealed tube applying drastic conditions led to isomerization of **143** to the respective 1,3-diene, Diels–Alder reaction, and subsequent Diels–Alder cycloreversion with loss of ethylene to furnish alternariol precursor **144**, which was then transferred to alternariol (**1**) with standard conditions.

**Scheme 9:**
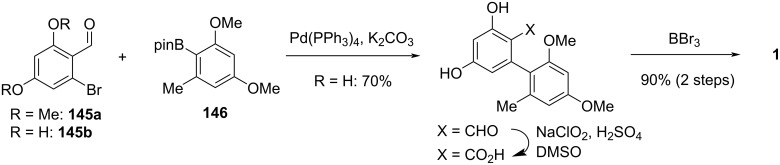
Total synthesis of alternariol (**1**) using a Suzuki strategy by Koch and Podlech [62], improved by Kim et al*.* [64].

The first competitive total synthesis, which allowed for the synthesis of significant amounts of the natural product (5 g were once synthesized in the author’s lab in one batch), was presented by Koch and Podlech (Scheme 9). It uses a Suzuki coupling as the key step [62]. Where this synthesis led to the formation of alternariol together with its 9-*O*-methyl ether **2**, which had to be separated by chromatography, a slight improvement presented by Kim et al. allowed for the preparation of alternariol without the formation of side products [64]. The originally proposed synthesis worked with dimethylated benzaldehyde **145a**. Suzuki coupling with boronate **146**, Krauss oxidation, and final deprotection led to alternariol containing about 20% AME (**2**), which had to be separated. When instead unprotected benzaldehyde **145b** was used for the respective route, final deprotection cleanly led to AOH without need for a laborious purification process.

The herein used strategy with a Suzuki coupling as key step was applied in a large number of further total syntheses for compounds presented in this review. An overview is given in Table 1.

**Table 1:** Total syntheses of alternariol-derived resorcylic lactones.

Compound	Key step^a^

alternariol (**1**)	A [65–68], B [62,64], C [63], E [34,61]
9-*O*-methylalternariol (**2)**	A [68], B [62]
3-*O*-methylalternariol (**3**)	A [66]
lysilactone A (**7**)	B [139–140], C [141]
graphislactone A (**21**)	B [44], C [166]
graphislactone B (**22**)	C [166]
graphislactone H (**23**)	B [44], C [177]
palmariol A (**25**)	C [179]
palmariol B (**26**)	C [179]
hyalodendriol C (**27**)	C [181]
graphislactone G (**28**)	B [182], C [179,183]
penicilliumolide D (**29**)	C [185]
altertenuol (**31**)	B [186], C [192]
graphislactone E (**32**)	B [44]
graphislactone F (**33**)	B [44]
autumnariol (**36**)	A [204], B [203], E [202]
autumnariniol (**37**)	E [202]
graphislactone C (**39**)	B [44], C [166]
graphislactone D (**40**)	B [44], C [166]
alterlactone (**41**)	B [217], E [218]
ulocladol (**42**)	B [44], C, D [220]
altenusin (**47**)	B [217,223–224]
desmethylaltenusin (**48**)	B [191]
decarboxyaltenusin (**50**)	B [247]
(−)-altenuene (**54**)	B [249]
(+)-isoaltenuene (**55**)	B [249]
(+)-neoaltenuene (**69**)	B [35]
*rac*-dehydroaltenusin (**74**)	B [223–224,277], C [277]
r*ac*-desmethyldehydroaltenusin (**75**)	B [191]
*rac*-TMC-264 (**79**)	E [185]
(+)-dehydroaltenuene A (**85**)	B, D [277]
*ent*-dehydroaltenuene B (**86**)	B, D [277]
(+)-dihydroaltenuene B (**92**)	B, D [277]
*rac*-altenuic acid III (**97**)	B [300]
altenuic acid IV (**98**)	B [301]
*rac*-cephalosol (**99**)	E [304–305]
*rac*-alternarlactam (**117**)	E [316]

^a^Biomimetic synthesis (A), Suzuki coupling (B), tethered biaryl formation (C), derivatization of a natural product (D), further methods (E).

**Scheme 10:**
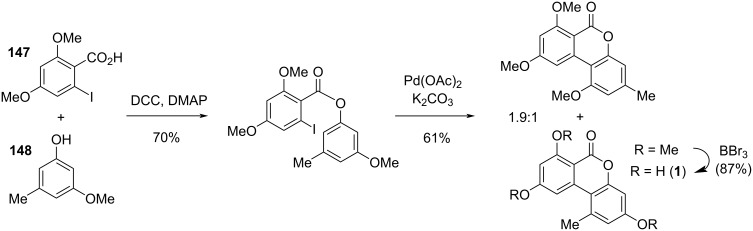
Total synthesis of alternariol (**1**) using an intramolecular biaryl coupling by Abe et al. [63].

Abe et al. presented a versatile method in which an esterification is followed by a tethered cross coupling. Where this strategy uses very simple and easily accessible precursors, it leads to constitutional isomers in scarce cases, which have to be separated. In the respective synthesis of alternariol, iodobenzoic acid **147** and orcinol derivative **148** were coupled in an esterification (using dicyclohexylcarbodiimide, DCC, and 4-dimethylaminopyridine, DMAP) and subjected to a palladium-catalyzed coupling (Scheme 10) [63]. The product mixture was separated and the methyl ether functions were cleaved to yield alternariol (**1**).

**Scheme 11:**
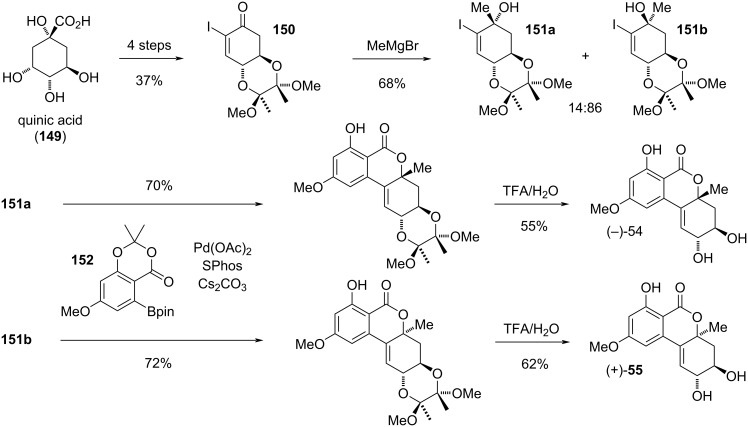
Total synthesis of altenuene (**54**) and isoaltenuene (**55**) by Podlech et al. [249].

In the total synthesis of altenuene (**54**) and isoaltenuene (**55**, Scheme 11), we started with iodinated cyclohexanone **150**, which was accessible from quinic acid (**149**) [249]. Addition of a methyl Grignard reagent furnished two tertiary alcohols **151a** and **b**, which were separated and individually reacted with boronate **152** in a Suzuki coupling, where concomitantly a lactonization occurred. Deprotection with trifluoroacetic acid (TFA) gave rise to altenuene and isoaltenuene. Both compounds were obtained as single enantiomers, where the respective chirality was similarly observed in some of the isolated natural products (vide supra).

**Scheme 12:**
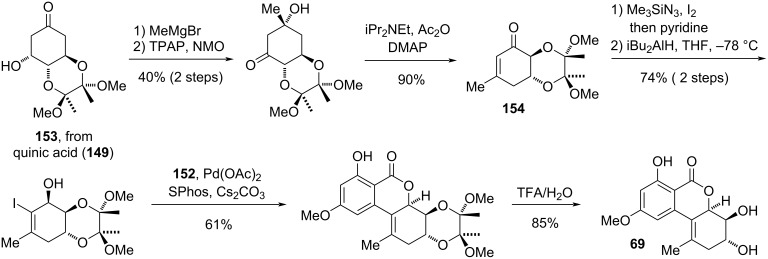
Total synthesis of neoaltenuene (**69**) by Podlech et al. [35].

Quinic acid (**149**) similarly served as the starting material in our synthesis of neoaltenuene (**69**) (Scheme 12) [35]. It was converted into protected trihydroxycyclohexanone **153**, which was subjected to a Grignard addition, oxidized (with tetrapropylammonium perruthenate, TPAP, and *N*-methylmorpholine *N*-oxide, NMO), and eliminated to enone **154**. (nota bene: The compound is now given in a different orientation.) Iodination and subsequent reduction of the keto function furnished an iodide suitable for Suzuki coupling with boronate **152**. The thus obtained lactone was deprotected to yield (+)-neoaltenuene with the here given absolute configuration. Nevertheless, no information on the chirality was given for the natural product [268].

**Scheme 13:**
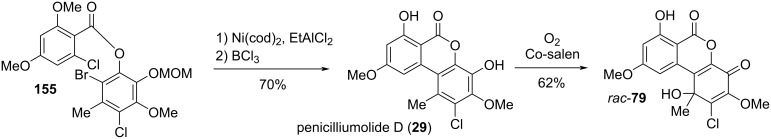
Total synthesis of TMC-264 (**79**) by Tatsuta et al. [185].

Total synthesis of TMC-264 (**79**) presented by Tatsuta et al. started with aryl benzoate **155**, which was easily obtained by esterification of suitable precursors (Scheme 13) [185]. Nickel-catalyzed intramolecular coupling to penicilliumolide D (**29**) and Co(salen)-catalyzed oxidation with molecular oxygen yielded TMC-264 as the racemate, which could be separated into the pure enantiomers by chiral HPLC.

**Scheme 14:**
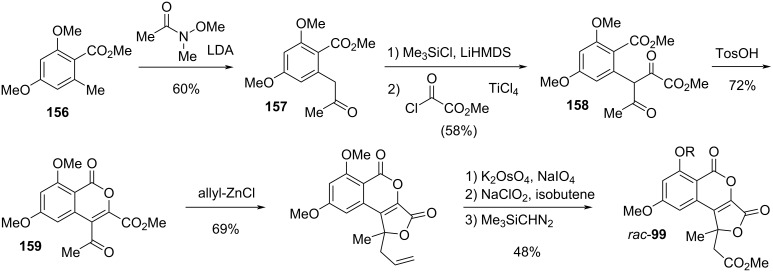
Total synthesis of cephalosol (**99**) by Koert et al. [304].

Hardly any total synthesis of the cyclopenta-fused resorcylic lactones has been published. Only the structures of the related altenuic acids III (**97**) [300] and IV (**98**) [301], of cephalosol (**99**, two total syntheses) [304–305], and of alternarlactam (**117**) [316] were confirmed by total syntheses of these compounds. The first total synthesis of cephalosol, published in 2010 [304], started with protected orsellinic acid **156**, which was deprotonated at the benzylic position and reacted with the Weinreb amide of acetic acid to yield **157** (Scheme 14). Mukaiyama aldol addition with monomethyl oxalyl chloride furnished a 1,3-dicarbonyl **158**, which was condensated to establish the resorcylic lactone moiety (**159**). Addition of an allyl Grignard reagent and transformation of the allylic double bond into an ester finalized the synthesis of racemic cephalosol (*rac*-**99**).

An overview on all published total syntheses of alternariol-derived resorcylic lactones is given in Table 1 together with notes on the used general synthetic strategy.

## Conclusion

The herein discussed resorcylic lactones derived from alternariol are a very divers class of compounds with far more than one hundred members. Although only about half a dozen of these natural products is found as fungal contaminants in food and feed and is thus of significant interest, e.g., in terms of food safety, most of the further identified metabolites show interesting biological properties and deserve further investigation. It would be very desirable if research in this field would be intensified and if studies on the biosynthesis similarly of the minor metabolites would be started. Further total syntheses could not only verify (or reject) the proposed structures but would supply sufficient material for these investigations. Hopefully, this review helped to get a comprehensive overview on this class of compounds supporting future work in this area.

## Supporting Information

File 1 Attempted synthesis of **38** and calculated ECD spectrum of **70**.

## Data Availability

All data that supports the findings of this study is available in the published article and/or the supporting information to this article.
