# Supplementary material for: Natural resorcylic lactones derived from alternariol
Source: Beilstein J Org Chem. 2024 Aug 30;20:2171–207. doi: 10.3762/bjoc.20.187 (PMC11368053; doi:10.3762/bjoc.20.187)
Supplement: File 1 — Attempted synthesis of 38 and calculated ECD spectrum of 70. [file Beilstein_J_Org_Chem-20-2171-s001.pdf]

BEILSTEIN JOURNAL OF ORGANIC CHEMISTRY

Supporting Information

for

Natural resorcylic lactones derived from alternariol

Joachim Podlech

Beilstein J. Org. Chem. **2024,** *20,* 2171–2207. doi:10.3762/bjoc.20.187

Attempted synthesis of 38 and calculated ECD spectrum of 70

License and Terms: This is a supporting information file under the terms of the Creative Commons Attribution License (https://creativecommons.org/licenses/by/4.0). Please note that the reuse, redistribution and reproduction in particular requires that the author(s) and source are credited and that individual graphics may be subject to special legal provisions.
The license is subject to the *Beilstein Journal of Organic Chemistry* terms and conditions: (https://www.beilstein-journals.org/bjoc/terms)

1. Attempted synthesis of a natural product 38 and comparison of NMR-spectroscopic data

2. Calculated ECD spectrum of hyalodendriol A (70)

1. Attempted synthesis of a natural product 38 and comparison of NMR-spectroscopic data

The total synthesis of the proposed structure of **38** (see Figure 11 in the publication) was finalized via the following the sequence given in the scheme.[1] NMR spectroscopic analysis of the thus obtained compound **SI-3** furnished NMR data, which do not agree with those published with the isolation of the natural product **38**.[2] The proposed structure of **38** is thus very likely not correct.

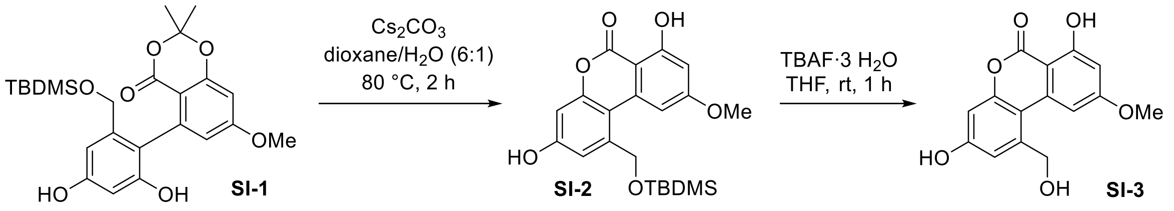

1-(*tert*-Butyldimethylsilyloxymethyl)-3,7-dihydroxy-9-methoxy-6*H*-benzo[*c*]chromen-6-one (SI-2):

Precursor **SI-1** (165 mg, 358 µmol) and Cs_2CO_3 (500 mg, 1.53 mmol) in dioxane/H_2O (6:1; 6 mL) were stirred for 2 h at 80 °C and cooled to rt. Saturated aqueous NH_4Cl solution was added and the mixture was extracted with EtOAc (3×). The combined organic layers were dried (Na_2SO_4), concentrated at reduced pressure, and purified by column chromatography (silica gel, CH_2Cl_2/MeOH, 100:2) to yield **SI-2** as an off-white solid (50 mg, 124 µmol, 35%). R_f = 0.50 (hexane/EtOAc, 2:1); ^{1}H NMR (400 MHz, DMSO-d$_6$): δ (ppm) = 11.75 (s, 1 H, OH), 10.58 (s, 1 H, OH), 7.24 (d, ^4J = 2.2 Hz, 1 H, ArH), 7.02 (d, ^4J = 2.7 Hz, 1 H, ArH), 6.71 (d, ^4J = 2.7 Hz, 1 H, ArH), 6.61 (d, ^4J = 2.1 Hz, 1 H, ArH), 4.98 (s, 2 H, CH_2), 3.89 (s, 3 H, OCH_3), 0.89 [s, 9 H, $C(CH_3)_3$], 0.12 (s, 6 H, 2×$SiCH_3$); ^{13}C NMR (101 MHz, DMSO-d$_6$): δ (ppm) = 166.4 (C), 164.6 (C), 164.0 (C), 159.0 (C), 152.5 (C), 140.5 (C), 136.6 (C), 115.2 (CH), 108.6 (C), 104.0 (CH), 102.8 (CH), 99.5 (CH), 98.5 (C), 64.6 (CH_2), 55.8 (OCH_3), 25.8 (3×CH_3), 18.0 (C), –5.1 (2×$SiCH_3$); IR (ATR): $\tilde{\nu}$ (cm^{-1}) = 3501 (vw), 2930 (w), 2854 (w), 1650 (m), 1585 (m), 1463 (m), 1388 (w), 1353 (m), 1249 (m), 1225 (m), 1202 (m), 1191 (m), 1047 (m), 998 (m), 983 (m), 885 (w), 832 (s), 795 (m), 777 (m), 741 (m), 690 (m), 638 (m), 621 (m); MS (ASAP): *m/z* (%) = 549.3 (35), 403.2 (100) $[M+1]^+$, 271.1 (3) $[M–C_6H_{15}OSi]^+$; HRMS: *m/z* $[M]^+$ calcd. for $C_{21}H_{27}O_6{}^{28}Si$: 403.1571; found: 403.1565.

3,7-Dihydroxy-1-(hydroxymethyl)-9-methoxy-6*H*-benzo[*c*]chromen-6-one (SI-3):

Precursor **SI-1** (50 mg, 109 µmol) and Cs_2CO_3 (200 mg, 614 µmol) in dioxane/H_2O (6:1; 2 mL) were stirred for 2 h at 80 °C and cooled to rt. Saturated aqueous NH_4Cl solution was added and the mixture was extracted with EtOAc (3×). The combined organic layers were dried (Na_2SO_4) and concentrated at reduced pressure to yield a crude product (40 mg), which was used without purification. It was dissolved in THF (5 mL), $Bu_4NF·3H_2O$-Lösung (1M in THF, 2 mL, 2.00 mmol) was added and the mixture was stirred for 1 h. Saturated aqueous NH_4Cl solution (20 mL) was added, stirring was continued for 10 min and the mixture

---

[1] Herzog, S; Podlech, J., unpublished work; Herzog, S., Totalsynthese eine neuen Resorcylsäurelactons. Master Thesis, Karlsruher Institut für Technologie (KIT), Karlsruhe, 2019.

[2] Zhang, J.-c.; Chen, G.-Y.; Li, X.-Z.; Hu, M.; Wang, B.-Y.; Ruan, B.-H.; Zhou, H.; Zhao, L.-X.; Zhou, J.; Ding, Z.-T.; Yang, Y.-B., *Nat. Prod. Res.* **2017**, *31*, 2745-2752, doi: 10.1080/14786419.2017.1295235.

was extracted with EtOAc (3×). The combined organic layers were dried (Na_2SO_4), concentrated at reduced pressure, and purified by column chromatography (silica gel, CH_2Cl_2/MeOH, 100:2) to yield **SI-3** as a white solid (8 mg, 28 µmol, 26%). ^{1}H NMR and ^{13}C NMR: see table; IR (ATR): $\tilde{\nu}$ (cm^{-1}) = 3211 (w), 1651 (m), 1613 (m), 1567 (m), 1449 (w), 1422 (w), 1355 (w), 1281 (w), 1224 (m), 1197 (m), 1161 (m), 1092 (m), 979 (m), 835 (w), 782 (w), 734 (w), 692 (w), 628 (m), 570 (w), 526 (w); MS (ASAP): *m/z* (%) = 289.1 (100) $[M+1]^+$; HRMS: *m/z* $[M]^+$ calcd. for $C_{15}H_{13}O_6$: 289.0707; found: 289.0702.

**Natural product (38)**[2]	**Synthesized compound (SI-3)**
^{13}C NMR (100 MHz, acetone-d_6)	^{13}C NMR (101 MHz, acetone-d_6)
56.9 (9-OMe)	56.2 (9-OMe)
70.7 (1-CH_2)	64.6 (1-CH_2)
102.2 (C-8)	99.9 (C-6a)
108.3 (C-6a)	100.4 (C-8)
109.2 (C-10)	103.7 (C-4)
116.7 (C-2)	105.1 (C-10)
117.7 (C-4)	110.7 (C-10b)
128.5 (C-10a)	116.6 (C-2)
132.8 (C-10b)	138.1 (C-10a)
143.2 (C-1)	142.7 (C-1)
147.6 (C-4a)	154.0 (C-4a)
148.2 (C-3)	159.8 (C-3)
165.0 (C-7)	165.6 (C-7)
165.6 (C-9)	166.0 (C-6)
174.3 (C-6)	167.9 (C-9)
^{1}H NMR (400 MHz, acetone-d_6)	^{1}H NMR (400 MHz, acetone-d_6)
3.96 (s, 3 H, 9-OMe)	3.96 (s, 3 H, 9-OMe)
5.13, 4.95 (br s, 2 H, 1-CH_2)	4.98 (d, 2 H, 1-CH_2)
6.58 (d, *J* = 1.6 Hz, 1 H, 8-H)	6.56 (d, 1 H, 8-H)
6.65 (d, *J* = 2.0 Hz, 1 H, 10-H)	6.77 (d, 1 H, 4-H)
7.07 (s, 1 H, 2-H)	7.14 (d, 1 H, 2-H)
7.16 (s, 1 H, 4-H)	7.53 (d, 1 H, 10-H)

2. Calculated ECD spectrum of hyalodendriol A (70)

Details on how this calculation was performed are given in a recent publication.[3] Six conformations with the lowest relative energies (all less than 10 kJ/mol) were considered and their ECD spectra were superimposed with Boltzmann weighting factors. The structure with the absolute configuration depicted in Figure 18 of the publication was used for calculation. Maxima and minima of the experimental ECD spectrum are included; a more meaningful and better comparable experimental full spectrum is given in the original publication.[4]

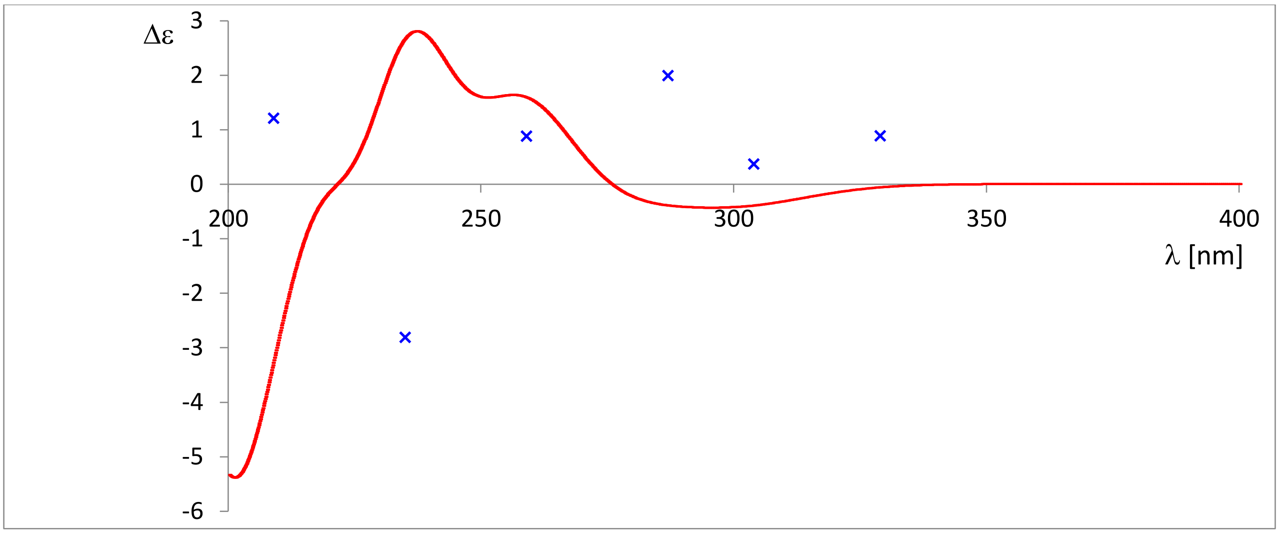

3 Podlech, J.; Gutsche, M., *J. Nat. Prod.* **2023**, *86*, 1632-1640, doi: 10.1021/acs.jnatprod.3c00078.

4 Mao, Z.; Lai, D.; Liu, X.; Fu, X.; Meng, J.; Wang, A.; Wang, X.; Sun, W.; Liu, Z. L.; Zhou, L.; Liu, Y., *Pest Manage. Sci.* **2017**, *73*, 1478-1485, doi: 10.1002/ps.4481.
